# A Systematic Review of Biomarkers for Disease Progression in Alzheimer's Disease

**DOI:** 10.1371/journal.pone.0088854

**Published:** 2014-02-18

**Authors:** David J. M. McGhee, Craig W. Ritchie, Paul A. Thompson, David E. Wright, John P. Zajicek, Carl E. Counsell

**Affiliations:** 1 Division of Applied Health Sciences, University of Aberdeen, Aberdeen, Scotland, United Kingdom; 2 Centre for Mental Health, Imperial College London, London, England, United Kingdom; 3 Centre for Health and Environmental Statistics, Plymouth University, Plymouth, England, United Kingdom; 4 Clinical Neurology Research Group, Plymouth University, Plymouth, England, United Kingdom; Emory University, United States of America

## Abstract

**Background:**

Using surrogate biomarkers for disease progression as endpoints in neuroprotective clinical trials may help differentiate symptomatic effects of potential neuroprotective agents from true slowing of the neurodegenerative process. A systematic review was undertaken to determine what biomarkers for disease progression in Alzheimer's disease exist and how well they perform.

**Methods:**

MEDLINE and Embase (1950–2011) were searched using five search strategies. Abstracts were assessed to identify papers meriting review in full. Studies of participants with probable Alzheimer's disease diagnosed by formal criteria were included. We made no restriction on age, disease duration, or drug treatment. We only included studies with a longitudinal design, in which the putative biomarker and clinical measure were both measured at least twice, as this is the only appropriate study design to use when developing a disease progression biomarker. We included studies which attempted to draw associations between the changes over time in the biomarker used to investigate disease progression and a clinical measure of disease progression.

**Results:**

Fifty-nine studies were finally included. The commonest biomarker modality examined was brain MRI (17/59, 29% of included studies). Median follow-up in included studies was only 1.0 (IQR 0.8–1.7) year and most studies only measured the putative biomarker and clinical measure twice. Included studies were generally of poor quality with small numbers of participants (median 31 (IQR 17 to 64)), applied excessively restrictive study entry criteria, had flawed methodologies and conducted overly simplistic statistical analyses without adjusting for confounding factors.

**Conclusions:**

We found insufficient evidence to recommend the use of any biomarker as an outcome measure for disease progression in Alzheimer's disease trials. However, further investigation into the efficacy of using MRI measurements of ventricular volume and whole brain volume appeared to be merited. A provisional ‘roadmap’ to improve the quality of future disease progression biomarker studies is presented.

## Introduction

The rising prevalence of Alzheimer's disease, the commonest neurodegenerative disorder, and the associated financial and social costs this brings, presents a major challenge to governments of countries with an ageing population [1]. Given that only symptomatic treatments for Alzheimer's disease currently exist, much research has focused on the development of drugs which slow or even halt neurodegeneration and, therefore, clinical progression. However, clinical trials in neurodegenerative disorders have struggled to separate out symptomatic effects of potential therapeutic agents (e.g. due to increased synaptic acetylcholine) from true disease-modifying effects. In Alzheimer's disease, it is currently not possible to directly measure the number of remaining cortical neurons in vivo and, therefore, alternative approaches are required. Clinical assessments in Alzheimer's disease using scales to measure cognitive impairment, disability, quality of life, or global disease severity are affected by symptomatic effects of therapy and are unable to differentiate this effect from disease-modification, at least in the short-term.

Various clinical trial designs have been developed to try to adjust for symptomatic effects of putative neurodegenerative agents and, therefore, allow clinical rating scales to be used as endpoints. These include long-term follow up studies of placebo-treated and active-agent treated patients looking for sustained divergence, measuring outcomes following a wash-out period, and delayed start trial designs [2]. However, analytic and logistical problems with these trial designs have as yet restricted their use [3]. An alternative approach, the focus of much primary research, is the use of a surrogate outcome biomarker as an endpoint in neuroprotective clinical trials.

Surrogate outcome biomarkers are objectively measured characteristics of a disease, which act as indicators of the underlying pathogenic process responsible for disease progression, including the change in that process following a therapeutic intervention [4], [5]. To allow their use in clinical trials surrogate outcome biomarkers must have a strong association with a clinical endpoint or outcome known to measure the effect of a therapeutic intervention on disease progression, for which the biomarker can act as a substitute. Surrogate biomarkers for disease progression in Alzheimer's disease could shorten the duration of phase III trials and thereby reduce the cost and time required to get a drug to market. Unfortunately at present there is not a single accepted surrogate outcome biomarker for any neurodegenerative disorder.

Much has been written about the features that a biomarker for disease progression in neurodegenerative disorders, such as Alzheimer's disease, should possess [6], [7]. The ideal surrogate biomarker should:

1. Change with neurodegeneration (i.e. degeneration of cortical neurons);
2. Show an association with the clinical phenotype arising secondary to this degenerative process;
3. Have a direct association with disease progression, without intermediate variables;
4. Have robust longitudinal data linking it to disease progression;
5. Not be influenced by symptomatic treatment, but only by a true change in the neurodegenerative process;
6. Predict long-term changes in disease progression by short-term changes in the biomarker;
7. Be generalisable to people with differing characteristics (e.g. age, gender, race);
8. Be continually variable (ideally linearly for simplicity);
9. Be sensitive, reflecting small changes in disease progression;
10. Be quick and cheap to measure, and amenable to blinded assessment;
11. Be suitable for measurement reliably across different centres;
12. Be suitable for repeated measurement in the same patient;
13. Be safe and tolerable to the patient.

As Alzheimer's disease is a complex neurodegenerative disorder in which many different pathophysiological processes have been implicated (e.g. tau and amyloid deposition, abnormalities of cholesterol metabolism, inflammation, oxidative damage and lysosomal dysfunction) it is not surprising that many different candidate biomarkers for disease progression in Alzheimer's disease have been studied. However, the literature in this area has never been brought together systematically. We, therefore, aimed to undertake a systematic review to assess what potential surrogate biomarkers for disease progression in Alzheimer's disease exist, whether any meet the criteria for use in clinical trials, and if not which looks most promising. We did not aim to review the literature for diagnostic biomarkers (i.e. those designed to aid early diagnosis in the pre-motor or motor phase) or prognostic biomarkers (i.e. those aimed at identifying patients who progress at different rates).

Given the methodological and statistical weaknesses we identified in studies of biomarkers for disease progression in Parkinson's disease (PD) in a previous systematic review [8], we aimed to determine whether the same problems were prevalent in Alzheimer's disease research. We, therefore, aimed to critique data from identified disease progression biomarker studies relating to study design, participant characteristics, and statistical analyses undertaken, in order to produce guidelines for future studies.

## Methods

Following the development of a review protocol, literature searches were conducted in the databases MEDLINE (1950 to November 2011) and Embase (1980 to November 2011), using the OVID search interface. Five separate search strategies, based on previous searches developed by an experienced information scientist, were run in each database. The first four were based on free-text words identified through background reading of relevant review articles. These searches included potential (1) blood, (2) urine or cerebrospinal fluid (CSF), (3) imaging and (4) neurophysiological biomarkers. A fifth search using generic terms for biomarkers based on index headings was also run in both databases. For details of the search strategy please see document S1.

The searches were limited to human studies. Only English language articles were included, due to lack of resources for translation. Reference lists of included articles and relevant review articles were checked to identify any studies which the electronic search strategy may have missed.

### Validation of the electronic search strategy

The electronic search strategy was validated by hand searching five years of the two journals from which most of the included articles came: Neurology (1995–1999) and Archives of Neurology (2001–2005). The number of relevant and irrelevant articles identified by hand searching and by the electronic search, was used to calculate the sensitivity and specificity for the electronic search strategy.

### Study selection

A single reviewer examined abstracts retrieved by the electronic search to identify articles meriting review in full. Full length articles were then reviewed before data were extracted from relevant papers. In both stages the inclusion and exclusion criteria detailed below were applied.

Only studies of participants with probable Alzheimer's disease diagnosed by formal criteria [9]–[13] were included. Studies which included participants with prodromal Alzheimer's disease or mild cognitive impairment (MCI) were only included if progression to Alzheimer's disease was confirmed in all participants by clinical follow-up. No restriction was made on the grounds of participant's age, disease duration, or drug treatment.

As emphasised in our previous systematic review of biomarkers for disease progression in PD [8], a cross-sectional study design, in which an association between a biomarker and a clinical measure of disease progression is examined at a single time point in a group of patients with differing disease severity, is not suitable to examine for a relationship between the change in a clinical measure and the change in a biomarker over time within individuals with a neurodegenerative disorder. We, therefore, limited this review to studies with a longitudinal design, where the biomarker and clinical measure were recorded at least twice.

Studies which investigated the efficacy of using a biomarker, including (but not restricted to) imaging, blood tests, tests of CSF and neurophysiological tests, to investigate disease progression in Alzheimer's disease were included. To qualify for inclusion there must have been an attempt to assess an association between the change in a biomarker and the change in a clinical measure of disease progression over time. Acceptable clinical measures included measures of cognitive impairment, disability, handicap, quality of life, and global clinical assessments.

Only studies exploring associations between a biomarker and the total score from a clinical rating scale, rather than its subsections, were included. The subsections of most clinical measures would not be acceptable outcome measures for neuroprotective trials and, therefore, developing surrogate biomarkers for them was not felt to be relevant. However, exceptions were made for the following clinical rating scale subsections, which may be acceptable outcome measures for disease-modification trials: Alzheimer's disease assessment scale cognitive (ADAS-cog) and non-cognitive (ADAS-noncog) subsections [14]; Blessed dementia scale change in performance of everyday activities subsection (Blessed ADL) [15]; CAMCOG memory subsection [16]. Similarly, only studies examining for associations between putative biomarkers and global measures of cognition, rather than individual neuropsychological tests were included. Furthermore, studies solely examining for associations between biomarkers and measures of neuropsychiatric impairment were not included, as depression and behavioural disturbance are not clearly associated with disease progression in Alzheimer's disease [17].

Studies examining the relationship between a biomarker and treatment status, the presence or severity of complications related to therapy, or duration of illness were excluded. We also excluded studies which examined for associations between symptomatic improvement, as measuring by clinical rating scales, and the change in the level or activity of cholinesterase enzymes in the blood or CSF following commencement of a cholinesterase inhibitor. As we wished to develop a biomarker for disease progression rather than a way of measuring the response to symptomatic therapy, these studies were not felt to be relevant.

### Data extraction

Study methods and results were extracted by a single reviewer, and to check for accuracy this was performed twice. Data were extracted, using a data extraction sheet (document S2) relating to the following: (1) study design including restrictiveness of criteria for entry into the study; (2) setting; (3) study population, including number of participants, gender ratio, disease duration at baseline, baseline measures of disease severity and baseline treatment status; (4) specific biomarkers investigated; (5) statistical analyses performed; (6) results of statistical analyses of the associations between the biomarkers and clinical measures of disease severity; (7) analysis of the effect of drug treatment on the biomarker; (8) economic analysis of using the biomarker; (9) measures of suitability and acceptability of the test to patients.

The restrictiveness of the inclusion and exclusion criteria applied to each study was graded as: none, explicit statement that only criteria to exclude other causes of dementia were applied; mild ≤3 criteria applied (except those described under moderate); moderate, 4–5 criteria applied or evidence of an attempt to limit by age, gender, cognitive state, drug therapy for Alzheimer's disease (e.g. all de-novo); severe≥6 criteria applied; not detailed, no mention of whether criteria were applied.

### Methodological quality

No validated tool to measure the quality of studies investigating surrogate biomarkers as outcome measures exists. An attempt was, therefore, made to assess study quality using a quality questionnaire developed in our previous systematic review of biomarkers for disease progression in PD (table 1).

**Table 1:** Quality criteria to assess studies examining surrogate biomarkers for disease progression[8].

	Question	Yes	No
(1)	Was the primary aim of the study to validate a biomarker for disease progression?	32	54
(2)	Did the study detail a scientifically valid reason for choosing the given biomarker for investigation?	59	100
(3)	Has the reproducibility of measuring the biomarker in the same centre by different trained personnel, and between centres, been evaluated?	2	3
(4)	Has an assessment of the effect of likely confounding factors (e.g. age, gender, smoking status, and being on symptomatic treatment) on the measurement of the biomarker been made?	1	2
(5)	Has an assessment of the validity and reliability of the criterion (e.g. clinical rating scale) used been made?	54	92
(6a)	Was a power calculation undertaken to determine the required number of participants?	3	5
(6b)	If a power calculation was undertaken, was the number of participants included appropriate?	1	2
(7)	Was the study longitudinal?	59	100
(8)	Was the study prospective?	49	83
(9)	Was there a sufficient period of follow-up?	26	44
(10)	Were the biomarker and clinical measures of disease severity measured on ≥3 occasions?	7	12
(11)	Was measurement of the biomarker blind to participant characteristics?	25	42
(12)	Did≥75% of participants entering the study complete the full follow-up period?	42	71
(13)	Were cases unselected/unbiased (no exclusion criteria)?	16	27
(14)	Were associations between the biomarker and clinical measures of disease severity examined for using appropriate statistical modelling (e.g. linear mixed modelling) with adjustment for confounding factors, rather than simply correlation analysis?	7	12
(15)	Were results of statistical analyses reported in sufficient detail to allow the inclusion of the study results in a meta-analysis?	14	24

Most articles did not provide information pertinent to question five (‘has an assessment of the validity and reliability of the criterion used been made?’), perhaps because it was assumed that readers would be aware of the psychometric properties of the criterion used. We, therefore, scored papers favourably for question five if they used a criterion examined in the review of outcome measures in clinical trials in Alzheimer's disease from the Canadian Coordinating Office for Health Technology Assessment (CCOHTA) [18]. Whilst the examination of the properties of a given clinical outcome measure in this review neither implies adequate or favourable psychometric assessment, it does at least indicate that some degree of psychometric assessment has occurred. Where more than one clinical rating scale was used to draw associations with a biomarker in a single paper, question five was marked favourably as long as at least one of the clinical measures was in the aforementioned review.

With regards to question nine (‘was there a sufficient period of follow-up?’) we denoted a sufficient period of follow-up in this review as longer than one year. Although this may be an insufficient period of follow-up to detect significant disease progression in Alzheimer's disease, we hoped this cut-off would at least help differentiate very short studies from those with longer periods of follow-up.

### Data analysis and synthesis

Given the likelihood that included studies would examine the relationship of multiple different putative biomarkers with multiple different clinical measures of disease severity, we were aware that any data synthesis would be qualitative in nature.

## Results

**Figure 1:**
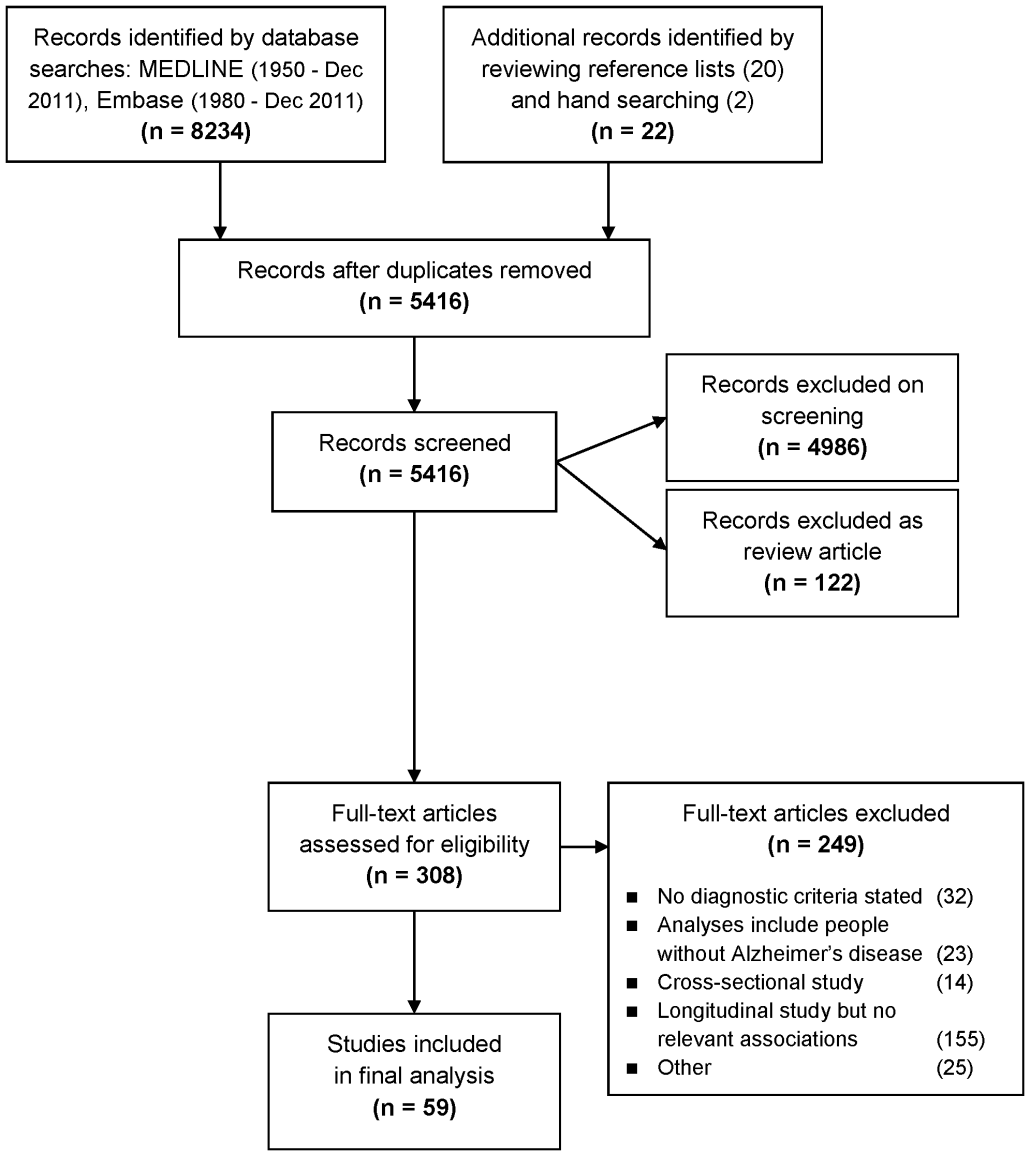
Flow diagram outlining the selection procedure to identify 59 articles which were included in the systematic review of biomarkers for disease progression in Alzheimer's disease. Note that of the 20 articles identified by reviewing reference lists, nine were excluded, and 11 were included in the final qualitative synthesis. Both articles identified by hand searching were included in the final qualitative synthesis.

As shown in Figure 1, the electronic searches identified 8234 records. After removing duplicates, 5416 unique records identified by the electronic search were screened, in addition to a further 22 records identified while performing the hand search or on reviewing reference lists of relevant review articles and included articles. The full-text articles of 308 records were then assessed for eligibility, and of those 249 articles were excluded. Finally data were extracted from a total of 59 articles.

### Hand searching

Hand searching to validate the electronic search strategy revealed a sensitivity of 60.0% (95% CI 17.0–92.7) and a specificity of 99.1% (95% CI 98.8–99.3). The number of included articles identified by the electronic search in both journals within the chosen time period was small (Archives of Neurology n = 2, Neurology n = 1). The low sensitivity related to the finding of one additional article in each journal on hand searching. However, both these articles had already been found prior to the hand search by searching the reference lists of included articles and review articles. Therefore, the actual sensitivity for the whole search process (electronic search plus review of reference lists) was higher (100.0% (95% CI 46.3–100.0)). Both articles were not identified by the electronic search as they lacked a term in their title relating to the biomarker modality used to examine for an association with a clinical measure of disease progression.

### Characteristics of included articles

All included studies except one made the diagnosis of Alzheimer's disease using the National Institute of Neurological and Communicative Disorders and Stroke-Alzheimer's Disease and Related Disorders Association (NINCDS-ADRDA) criteria [9], [10]. In two studies the diagnosis was, in at least some participants, confirmed using neuropathological diagnostic criteria [19], [20].

As illustrated in table 2, almost half of the included studies did not describe their setting, but the vast majority of those who did were based in outpatient departments. Similarly, almost a third of studies failed to mention whether inclusion and exclusion criteria were applied. Of those providing this information more than three quarters applied moderately to severely restrictive study entry criteria.

**Table 2:** Study characteristics of the 59 included articles and baseline characteristics of participants with Alzheimer's disease included in those studies.

**Setting of included studies**		
Outpatient	29	49%
Outpatients and inpatients	1	2%
Inpatient	1	2%
Not detailed	28	47%
**Inclusion/exclusion criteria applied in included studies**		
None	0	0%
Mildly restrictive	5	9%
Moderately restrictive	21	36%
Severely restrictive	15	26%
Not detailed	17	29%
**Baseline demographics**		
Median number of patients	31	(17 to 64)
Mean age (years)	73.0	(4.0)
Mean percentage male	42	(14)
Median disease duration (years)	3.6	(2.9 to 4.3)
Median percentage treated with a cognitive enhancer	0	(0 to 73)
**Baseline disease severity**		
Median MMSE	21	(20 to 23)

The number and percentage of included studies with each study characteristic is presented. Means are presented with standard deviations, and medians with interquartile ranges (IQR).

All of the included studies used an impairment or disability scale as the clinical measure of disease progression used to test for an association with a biomarker. None of the studies used measures of quality of life or handicap as a clinical outcome measure.

### Characteristics of study participants

As illustrated in table 2, the median number of study participants was low at 31 (interquartile range (IQR) 17 to 64). The mean age of those included was fairly young at 73.0 (standard deviation (SD) 4.0) years of age, particularly considering that the median duration of disease at study entry was 3.6 (IQR 2.9 to 4.3) years.

The majority of participants were not on a cognitive enhancer at baseline and had mild dementia, as assessed by the MMSE. Unfortunately, insufficient numbers of studies quoted participants' baseline scores on other widely used cognitive rating scales to allow meaningful descriptive statistics relating to these measures to be calculated.

### Quality criteria

The median total score produced by applying the quality questionnaire to each of the included studies was 7.0 (IQR 6.0 to 7.0) out of a possible 16 (table 1). There was no evidence to suggest that the quality scores achieved for recently published studies were better than for those published in the past. In just over half of the included studies the primary aim was to develop a biomarker for disease progression. Whilst all studies were rated as having given a valid reason for choosing the biomarker in question for investigation, this question was difficult to score for studies whose primary aim was not to develop a biomarker for disease progression. In those cases credit was given for a reasonable explanation of why the studies true aims were scientifically credible.

The vast majority of studies did not describe the reproducibility of measuring the biomarker, even in a single centre, and in most cases no details of the effects of confounding factors on the biomarker under investigation were described. The majority of studies did, however, use at least one clinical rating scale examined in the CCOHTA review. Only three studies undertook a power calculation to determine the number of participants, and only one of these recruited the required number of participants.

The median length of follow-up was only 1.0 (IQR 0.8 to 1.7) years, and most studies only measured the putative biomarker and clinical measure of disease severity twice (mean number of time-points 2.3 (SD 0.9)). Unfortunately in a few studies it was impossible to ascertain exactly how long participants were followed-up, or how many measurements were taken. Over half of the included studies also failed to state whether measurement of the biomarker was undertaken by an operator blind to the participants' characteristics.

In most studies over 75% of those entering the study at baseline completed the follow-up period. However, in many cases it appeared that analyses were restricted to a select cohort of patients, drawn from a larger unspecified cohort, who had completed the study period and, therefore, the true drop-out rate was probably higher.

### Types of biomarkers investigated

The biomarker modalities examined in the 59 included studies are summarised in table 3, along with details of whether or not a significant association between each biomarker modality and a clinical measure of disease progression was demonstrated in each study. Full details of the individual biomarkers examined in each study and their relationship with clinical measures of disease severity are given in tables S1 to S9.

**Table 3:** Comparison of the number of included studies investigating a given biomarker modality with the number reporting a significant association between the biomarker modality and a clinical measure of disease progression.

Biomarker modality	Number of studies investigating biomarker modality	Number of studies reporting a significant association between biomarker modality and a clinical measure of disease progression
Brain MRI	17 (29%)	14
CSF	12 (20%)	4
Brain MRS	8 (14%)	8
Serum/plasma/blood	7 (12%)	4
Brain PET	6 (10%)	4
Brain SPECT	5 (9%)	4
Electrophysiology	4 (7%)	3
Brain CT	1 (2%)	1
Ultrasound	1 (2%)	1

Two studies examined for a relationship between two different biomarker modalities (MRI and MRS in one study; MRI and CSF in another study) and a clinical measure of disease progression. (MRI, magnetic resonance imaging; CSF, cerebrospinal fluid; MRS, magnetic resonance spectroscopy; PET, positron emission tomography; SPECT, single-photon emission computed tomography; CT, computed tomography).

Multiple different candidate biomarkers for disease progression have been investigated in Alzheimer's disease, but the majority of studies examined some form of brain imaging. Those studies which reported a significant relationship were generally single studies examining a specific brain region, electrophysiological feature, blood or CSF constituent, with results which had not been replicated by other groups. In addition they involved small numbers of participants. The only biomarkers which appeared to have sufficient evidence to merit further investigation on the basis of the evidence presented in tables S1 to S9 were measures of ventricular volume (associations with MMSE and ADAS-cog) and whole brain volume (associations with MMSE) made using magnetic resonance imaging (MRI).

No studies reported an economic analysis of using the biomarker in question, and nor did any report on the acceptability of the test to individual patients. Six studies did, to some extent, examine the effect of symptomatic drug therapy on the biomarker under examination.

### Statistics

Correlation analysis, a basic statistical method which can be used to examine for a relationship between two variables, was solely used in 76% (37/49) of the included studies in which a description of the statistical techniques used was provided. Ten of the 59 included studies (17%) failed to report what statistical techniques were employed. Only two studies used a mixed model, despite the advantages this technique conveys in terms of dealing with missing data. Only seven (12%) of the included studies made adjustments for confounding factors. Furthermore, only 14 (24%) fully reported the outcome of their statistical analyses. Even when basic correlation analyses were conducted, correlation coefficients and significance values were often not reported and in no case were confidence intervals for the correlation coefficient given.

## Discussion

We found insufficient evidence to support the current use of any biomarker to measure disease progression in Alzheimer's disease clinical trials. Measurements of ventricular volume and whole brain volume made by MRI do, however, appear to merit further investigation.

**Figure 2:**
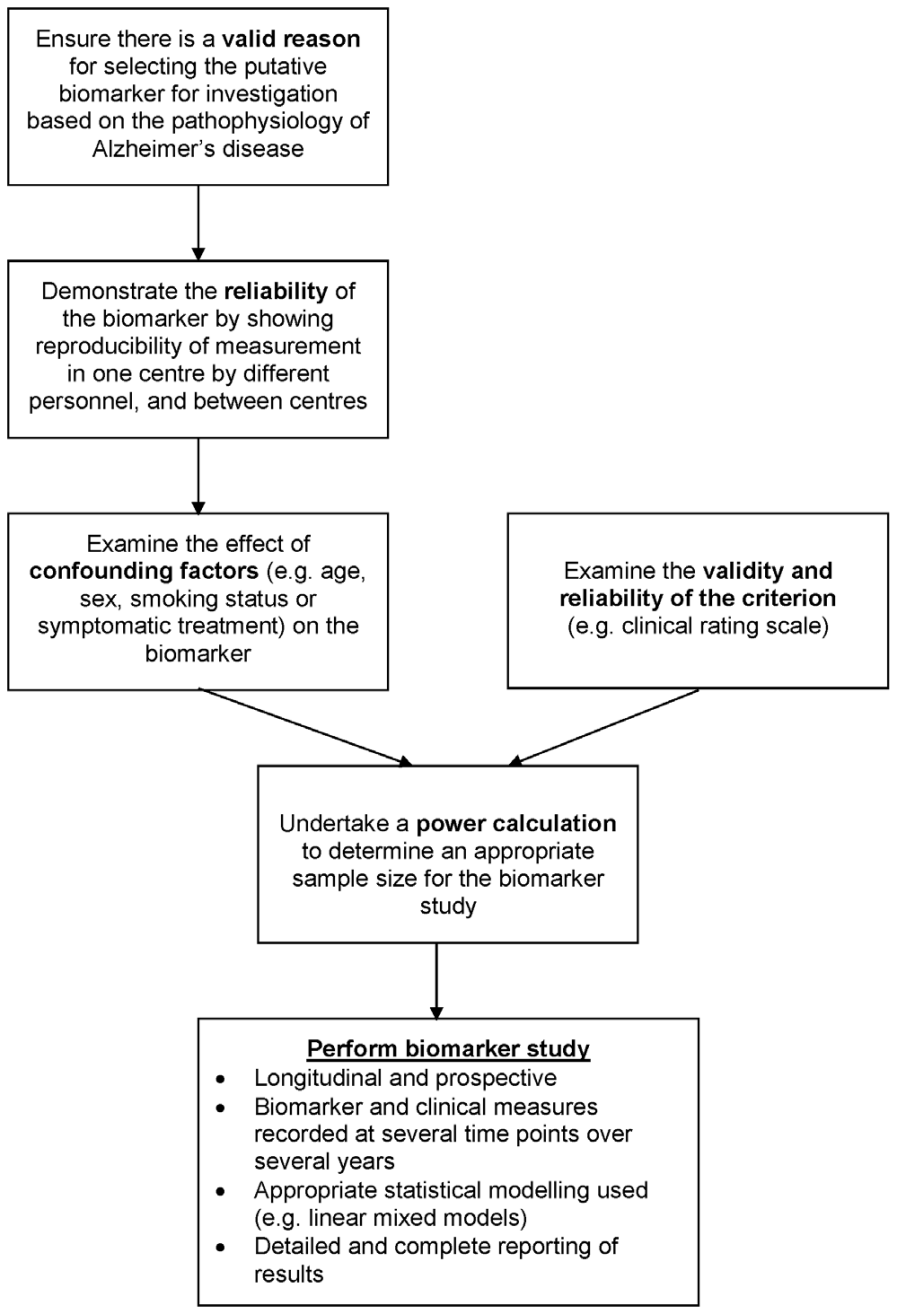
Flow diagram outlining a provisional ‘roadmap’ for conducting a study to determine whether a given biomarker is a suitable surrogate for a clinical measure of disease progression.

It is possible that the lack of a current biomarker in Alzheimer's disease is because no suitable biomarker exists or, at least, no single biomarker given the complexity of the disease. However, in keeping with the findings of our previous systematic review in PD [8], at present, it probably also reflects the poor quality of studies which have investigated biomarkers for disease progression. In order to improve future studies we previously developed a provisional ‘roadmap’ for conducting biomarker studies primarily in PD (Figure 2) but this ‘roadmap’ clearly also applies to Alzheimer's disease and other neurodegenerative diseases.

The starting point for any disease progression biomarker study must be a valid reason for selecting a specific biomarker for investigation based on the pathophysiology of the disease in question. Unfortunately, the development of a biomarker was not the primary aim of several studies included in this review; relevant analyses were simply the by-product of studies with an alternative aim (e.g. drug development). The appropriateness of studies with an alternative primary aim undertaking additional analyses to produce information regarding such associations is questionable. As our ‘roadmap’ highlights biomarker studies require careful planning and, therefore, should only run alongside other types of studies (e.g. long-term prognostic studies or clinical trials) when either such planning has taken place or as part of the process of gathering specific preparatory data required for a future formal biomarker study. Whilst this systematic review could be criticised for including studies whose primary aim was not to develop a biomarker for disease progression in Alzheimer's disease, we did so to ensure our review was as inclusive as possible and to avoid missing any potential biomarkers.

Secondly, the reliability of a putative biomarker must be established by demonstrating the reproducibility of its measurement in a single centre by different personnel, and between different centres. With imaging biomarkers characterised by a small change in a small area of the brain reliability of measurement can be a real issue, particularly between different centres which may have different imaging equipment and software.

Thirdly, an evaluation of the effect of potential confounding factors on the biomarker (e.g. age, gender, smoking status or cognitive enhancers) should be undertaken. An understanding of the influence of these factors on the biomarker will aid sample size calculations, and allow a rigorous analysis of the final study results by adjusting for these factors.

In parallel to this pre-study ‘work-up’ of the biomarker the validity, reliability, and responsiveness, including to clinical change, of the selected criterion against which a biomarker will be examined, must be explored. Extensive work has been undertaken in assessing the validity and reliability of psychometric instruments [21], and a similar approach here would seem sensible. Maximising the scientific rigor of the selected criterion is central to improving the chance of coming to the correct conclusion about the efficacy of a biomarker for disease progression, and will have implications for biomarker study sample size calculations.

Following these initial steps it should then be possible to perform a power calculation to determine an appropriate sample size before a biomarker study commences. Sample sizes can be adjusted to accommodate potential losses to follow-up which, as in the studies included in this review, are commonly encountered in longitudinal studies. However, only three studies in this review performed a power calculation, and only one of these then went on to recruit sufficient participants. Moreover, the small number of participants (median 31 (IQR 17 to 64)) in the studies included in this review is of concern. As studies become smaller it is increasingly likely that potentially significant associations will not be detected, and the number of variables which can be included in multivariate analyses without significantly increasing the risk of spurious findings becomes limited.

Whilst we only included longitudinal studies in this review it was clear from filtering the abstracts returned by the electronic search that, as in PD, numerous cross-sectional disease progression biomarker studies have been performed in Alzheimer's disease. As already discussed, this is not a suitable design to examine for a relationship between a change in a clinical measure and the change in a biomarker over time within individuals with Alzheimer's disease. The studies included in this review had a median follow-up duration of only 1.0 (IQR 0.8 to 1.7) years, with only 44% of studies following participants up for longer than our chosen discriminator of one year. There is currently no evidence to suggest what the minimum duration of a disease progression biomarker study should be, but it obviously needs to be long enough for a clinically significant change in the criterion, used to draw associations with the putative biomarker, to be observed. However, if a short-term change in a biomarker is to be associated with a long-term change in a clinical outcome measure then clearly a longer period of follow-up is required. In the included studies the biomarker and clinical measures were generally only measured twice (mean 2.3 (SD 0.9) time points). This is clearly insufficient to allow a linear association to be differentiated from a non-linear association. Future studies in this area must be longitudinal and measure the biomarker and clinical measures at several time points (at least three) over a sufficient follow-up period, more likely to be measured in years than months, as only this design will provide sufficient evidence of a biomarkers potential validity.

The use of moderately to severely restrictive entry criteria in the majority of studies included in this review will clearly have influenced the participants' characteristics. In particular, the elderly appear to be underrepresented in the included studies. One American incidence study, for example, found that whilst Alzheimer's disease had an annual incidence of 280 per 100,000 in those aged 65–69 years of age this rose dramatically to 5610 per 100,000 in those over 90 years of age [22]. Similar results have been reported in other American and European studies [23], [24]. This makes the mean age of 73.0 (SD 4.0) years of age of participants in the studies included in this review concerning, particularly as the median duration of disease at study entry was 3.6 (IQR 2.9 to 4.3) years. We would recommend that future studies try to keep their entry criteria as open as possible to maximise the generalisability of their results.

Reporting of statistical analyses in the included studies was inadequate. In both correlation and regression analyses, hypothesis testing can be undertaken to determine whether a relationship exists in the population as a whole, and confidence intervals calculated to indicate the strength of that relationship. Whilst all included studies undertook significance testing many failed to report precise significance values, and instead gave results descriptively in the text. Whilst this may reflect pressures of space in published journals, the results should at least be provided as a supplementary online resource. Several studies unfortunately even failed to detail what statistical techniques they used. Without clear reporting of the study methodology, results, and the outcome of statistical analyses, investigators devalue their study and risk it being excluded from future systematic reviews or meta-analyses.

The statistical techniques applied in the included studies were in several cases inappropriate and, more often than not, too simplistic. There was an overreliance on correlation, which is a limited technique to examine for a relationship between the changes in two variables as it only indicates the strength and direction of a relationship, and does not allow adjustment for confounding factors [25]. There was a tendency in the included articles for multiple individual correlation coefficients and significance values to be calculated after measuring a large number of variables rather than using a multivariate analysis or a higher level of statistical modelling (e.g. a linear mixed model [26]). The majority of studies also failed to adjust for important confounding factors, regardless of what statistical techniques they used.

We encountered the same deficiencies in statistical methodologies in the articles included in our previous systematic review of biomarkers for disease progression in PD [8], and in that paper discussed at length potential solutions to these problems. We strongly recommend that future biomarker studies incorporate a range of analyses, rather than simply correlation, in order to explore the validity of more advanced statistical methods. Using appropriate statistical techniques should reduce the chance of type I and type II errors and, thereby, allow sensible conclusions to be drawn about the efficacy of specific biomarkers. Analyses should be planned and conducted by an experienced statistician given the complexities of dealing with repeated measures data.

It is pleasing to note that some of the lessons of this systematic review have already begun to be realised by some researchers and put into practice. The longitudinal Alzheimer's Disease Neuroimaging Initiative (ADNI) [27] aims to measure various putative CSF and imaging biomarkers several times over several years. Their work, like that of the Parkinson's Progression Markers Initiative (PPMI) [28] and the Parkinson's Disease Biomarkers Program (PDBP) [29], should mark a major shift in the quality of studies of biomarkers for disease progression, and hopefully lead to advances in this important field.

## Conclusions

This extensive systematic review found insufficient evidence to recommend the use of any biomarker for measuring disease progression in Alzheimer's disease clinical trials. However, further examination of the efficacy of MRI measurements of ventricular volume and whole brain volume as biomarkers of disease progression in Alzheimer's disease does appear to be merited. As in our previous systematic in PD, we found methodological, statistical and reporting flaws in studies examining disease progression in Alzheimer's disease. Our methodological guidelines should hopefully provide a better chance of making progress in this area, and we would value feedback on them.

## Supporting Information

Document S1 **Electronic search strategy.**
(DOCX)

Document S2 **Data extraction sheet.**
(DOCX)

Table S1 **Blood, plasma and serum biomarkers.**
(DOCX)

Table S2 **CSF biomarkers.**
(DOCX)

Table S3 **Ultrasound biomarkers.**
(DOCX)

Table S4 **CT brain biomarkers.**
(DOCX)

Table S5 **Brain MRI biomarkers.**
(DOCX)

Table S6 **Brain MRS biomarkers.**
(DOCX)

Table S7 **Brain SPECT biomarkers.**
(DOCX)

Table S8 **Brain PET biomarkers.**
(DOCX)

Table S9 **Electrophysiological biomarkers.**
(DOCX)

Checklist S1 **PRISMA checklist.**
(DOC)
