# Correction: A Systematic Review of Biomarkers for Disease Progression in Alzheimer's Disease

**DOI:** 10.1371/journal.pone.0097960

**Published:** 2014-05-15

**Authors:** The PLOS ONE Staff

There is an error in Table 1. The column headings are incorrectly listed as "Question", "Yes" and "No". The column headings should be "Question", "Number" and "%". You will find an updated version of Table 1 below.

**Table 1:** Quality criteria to assess studies examining surrogate biomarkers for disease progression[8].

	Question	Number	%
(1)	Was the primary aim of the study to validate a biomarker for disease progression?	32	54
(2)	Did the study detail a scientifically valid reason for choosing the given biomarker for investigation?	59	100
(3)	Has the reproducibility of measuring the biomarker in the same centre by different trained personnel, and between centres, been evaluated?	2	3
(4)	Has an assessment of the effect of likely confounding factors (e.g. age, gender, smoking status, and being on symptomatic treatment) on the measurement of the biomarker been made?	1	2
(5)	Has an assessment of the validity and reliability of the criterion (e.g. clinical rating scale) used been made?	54	92
(6a)	Was a power calculation undertaken to determine the required number of participants?	3	5
(6b)	If a power calculation was undertaken, was the number of participants included appropriate?	1	2
(7)	Was the study longitudinal?	59	100
(8)	Was the study prospective?	49	83
(9)	Was there a sufficient period of follow-up?	26	44
(10)	Were the biomarker and clinical measures of disease severity measured on ≥3 occasions?	7	12
(11)	Was measurement of the biomarker blind to participant characteristics?	25	42
(12)	Did ≥ 75% of participants entering the study complete the full follow-up period?	42	71
(13)	Were cases unselected/unbiased (no exclusion criteria)?	16	27
(14)	Were associations between the biomarker and clinical measures of disease severity examined for using appropriate statistical modelling (e.g. linear mixed modelling) with adjustment for confounding factors, rather than simply correlation analysis?	7	12
(15)	Were results of statistical analyses reported in sufficient detail to allow the inclusion of the study results in a meta-analysis?	14	24
