# Supplementary material for: A Systematic Review of Biomarkers for Disease Progression in Alzheimer's Disease
Source: PLoS One. 2014 Feb 18;9(2):e88854. doi: 10.1371/journal.pone.0088854 (PMC3928315; doi:10.1371/journal.pone.0088854)
Supplement: Document S1 — Electronic search strategy. (DOCX) [file pone.0088854.s001.docx]

# *Additional document S1: Electronic search strategy*

**Systematic review of biomarkers for disease progression in Alzheimer’s disease**

## 1 Alzheimer’s disease and Blood (MEDLINE and Embase)

1. alzheimer*.ti. or exp *alzheimer disease/
2. (blood or plasma or serum or lymphocyte* or leucocyte* or leukocyte*).ti.
3. exp *amyloid/
4. (interleukin* or IL-1* or IL-6 or IL-8 or IL-10 or IL-12 or TNF* or tumour-necrosis factor* or tumor-necrosis factor*).ti.
5. (free radical* or reactive oxygen species or reactive nitrogen species).ti.
6. glutamine synthetase.ti.
7. (heme oxygensae* or haem oxygenase* or haeme oxygenase*).ti.
8. biliverdin reductase*.ti.
9. (copper or zinc).ti.
10. progranulin.ti.
11. (BACE1 or beta-site app-cleaving enzyme).ti.
12. (3-nt or 3-nitrotyrosine).ti.
13. (hne or 4-hydroxy-2-nonenal).ti.
14. prosaposin.ti.
15. cholesterol.ti.
16. (dhea or dehydroepiandrosterone).ti.
17. sulfatide*.ti.
18. s100*.ti.
19. (low-density lipoprotein receptor-related protein-1 or lrp1).ti.
20. (protein kinase* or mapk*).ti.
21. (mrna or messenger rna).ti.
22. (metallopeptidase* or metalloprotease*).ti.
23. kallikrein*.ti.
24. (IGF* or insulin-like growth factor*).ti.
25. phospholipase.ti.
26. 2 or 3 or 4 or 5 or 6 or 7 or 8 or 9 or 10 or 11 or 12 or 13 or 14 or 15 or 16 or 17 or 18 or 19 or 20 or 21 or 22 or 23 or 24 or 25
27. 1 and 26
28. (prognos* or progression).tw.
29. exp prognosis/
30. exp disease progression/
31. exp biological markers/
32. (biomarker* or bio-marker* or marker*).tw.
33. exp epidemiologic studies/
34. 28 or 29 or 30 or 31 or 32 or 33
35. 27 and 34
36. (cohort or prospective or retrospective or follow-up or longitudinal).tw.
37. cross-sectional.tw. or cross-sectional/
38. 36 and 37
39. 37 not 38
40. 35 not 39
41. (mouse or mice or murine or rat or rats or animal or rodent or monkey or primate or drosophila).ti.
42. 40 not 41
43. limit 42 to yr="1980 -Current"
44. remove duplicates from 43

**Total retrieved = 2458**

## 2 Alzheimer’s disease and Urine or CSF

## (MEDLINE and Embase)

1. alzheimer*.ti. or exp *Alzheimer disease/
2. urine.ti.
3. (NTP or neural thread protein).ti.
4. (cerebrospinal fluid or CSF).ti.
5. exp *amyloid/
6. (BACE or beta-site app-cleaving enzyme).ti.
7. exp *tau Proteins/
8. transferrin.ti.
9. glycan*.ti.
10. s100*.ti.
11. (acetylcholinesterase or AChE).ti.
12. kallikrein*.ti.
13. (PEDF or pigment epithelium-derived factor).ti.
14. (Interleukin* or IL-1 or IL-6 or TNF* or tumor-necrosis factor* or tumour-necrosis factor*).ti.
15. (complement or C3*).ti.
16. (isoprostane adj f2alpha).ti.
17. isoprostane.ti.
18. advanced glycation end products.ti.
19. thp.ti.
20. ykl-40.ti.
21. fibrinogen.ti.
22. (inos or nitric oxide synthetase).ti.
23. glutamate.ti.
24. 2 or 3 or 4 or 5 or 6 or 7 or 8 or 9 or 10 or 11 or 12 or 13 or 14 or 15 or 16 or 17 or 18 or 19 or 20 or 21 or 22 or 23
25. 1 and 24
26. (prognos* or progression).tw.
27. exp prognosis/
28. exp disease progression/
29. exp biological markers/
30. (biomarker* or bio-marker* or marker*).tw.
31. exp epidemiologic studies/
32. 26 or 27 or 28 or 29 or 30 or 31
33. 25 and 32
34. (cohort or prospective or retrospective or follow-up or longitudinal).tw.
35. cross-sectional.tw. or cross-sectional/
36. 34 and 35
37. 35 not 36
38. 33 not 37
39. (mouse or mice or murine or rat or rats or animal or rodent or monkey or primate or drosophila).ti.
40. 38 not 39
41. limit 40 to yr="1980 -Current"
42. remove duplicates from 41

**Total retrieved = 2755**

## 3 Alzheimer’s disease and Imaging (MEDLINE and Embase)

1. alzheimer*.ti. or exp *alzheimer disease/
2. imaging.ti.
3. neuroimaging.ti.
4. (mri or magnetic resonance).ti.
5. (ct or tomography).ti.
6. (pet or spect).ti.
7. (magnetoencephalography or MEG).ti.
8. (pittsburg compound b or PiB*).ti.
9. (fluorodeoxyglucose or fdg).ti.
10. fddnp.ti.
11. (spectroscopy or MRS).ti.
12. (morphometry or TBM).ti.
13. florbetapir.ti.
14. hmpao.ti.
15. (naa or n-acetyl aspartate).ti.
16. (creatine or choline).ti.
17. (regional cerebral blood flow or rcbf).ti.
18. (regional cerebral glucose metabolism or rcmrglu).ti.
19. 2 or 3 or 4 or 5 or 6 or 7 or 8 or 9 or 10 or 11 or 12 or 13 or 14 or 15 or 16 or 17 or 18
20. 1 and 19
21. (prognos* or progression).tw.
22. exp prognosis/
23. exp disease progression/
24. exp biological markers/
25. (biomarker* or bio-marker* or marker*).tw.
26. exp epidemiologic studies/
27. 21 or 22 or 23 or 24 or 25 or 26
28. 20 and 27
29. (cohort or prospective or retrospective or follow-up or longitudinal).tw.
30. cross-sectional.tw. or cross-sectional/
31. 29 and 30
32. 30 not 31
33. 28 not 32
34. (mouse or mice or murine or rat or rats or animal or rodent or monkey or primate or drosophila).ti.
35. 33 not 34
36. limit 35 to yr="1980 -Current"
37. remove duplicates from 36

**Total retrieved = 1265**

## 4 Alzheimer’s disease and Neurophysiology

## (MEDLINE and Embase)

1. alzheimer*.ti. or exp *alzheimer disease/
2. (emg or electromyography).ti.
3. (eeg or electroencephalography).ti.
4. electrophysiolog*.ti.
5. proteomics.ti.
6. metabolomics.ti.
7. neurochemical.ti.
8. transcriptomics.ti.
9. 2 or 3 or 4 or 5 or 6 or 7 or 8
10. 1 and 9
11. (prognos* or progression).tw.
12. exp prognosis/
13. exp disease progression/
14. exp biological markers/
15. (biomarker* or bio-marker* or marker*).tw.
16. exp epidemiologic studies/
17. 11 or 12 or 13 or 14 or 15 or 16
18. 17 and 10
19. (cohort or prospective or retrospective or follow-up or longitudinal).tw.
20. cross-sectional.tw. or cross-sectional/
21. 19 and 20
22. 20 not 21
23. 18 not 22
24. (mouse or mice or murine or rat or rats or animal or rodent or monkey or primate or drosophila).ti.
25. 23 not 24
26. limit 25 to yr="1980 -Current"
27. remove duplicates from 26

**Total retrieved = 209**

## 5. Alzheimer’s disease and Biomarkers

### 5.1 MEDLINE

1. *alzheimer disease/bl, cf, ri, us, ur
2. (prognos* or progression).tw.
3. *prognosis/
4. exp disease progression/
5. 2 or 3 or 4
6. 1 and 5
7. exp alzheimer disease/
8. alzheimer*.ti.
9. 7 or 8
10. exp biological markers/
11. (biomarker* or bio-marker* or marker*).ti.
12. 10 or 11
13. exp disease progression/
14. progression.tw.
15. 13 or 14
16. 9 and 12 and 15
17. 6 or 16
18. (cohort or prospective or retrospective or follow-up or longitudinal).tw.
19. cross-sectional.tw. or cross-sectional/
20. 18 and 19
21. 19 not 20
22. 17 not 21
23. (mouse or mice or murine or rat or rats or animal or rodent or monkey or primate or drosophila).ti.
24. 22 not 23
25. limit 24 to yr="1980 -Current"
26. Remove duplicates from 25

**Total retrieved = 669**

### 5.2 Embase

1. exp *alzheimer Disease/
2. alzheimer*.ti.
3. 1 or 2
4. exp Biological Markers/
5. (biomarker* or marker* or bio-marker*).m_titl.
6. 4 or 5
7. Disease course/
8. progression.tw.
9. 7 or 8
10. 3 and 6 and 9
11. exp longitudinal study/
12. exp follow up/
13. (progression or prognos*).tw.
14. 11 or 12
15. 2 and 13 and 14
16. 10 or 15
17. (cohort or prospective or retrospective or follow-up or longitudinal).tw.
18. cross-sectional.tw. or cross-sectional/
19. 17 and 18
20. 18 not 19
21. 16 not 20
22. (mouse or mice or murine or rat or rats or animal or rodent or monkey or primate or drosophila).ti.
23. 21 not 22
24. limit 23 to yr="1980 -Current"
25. Remove duplicates from 24

**Total retrieved = 878**
