# Supplementary material for: A Systematic Review of Biomarkers for Disease Progression in Alzheimer's Disease
Source: PLoS One. 2014 Feb 18;9(2):e88854. doi: 10.1371/journal.pone.0088854 (PMC3928315; doi:10.1371/journal.pone.0088854)

# *Additional document S2: Data extraction sheet*

**Systematic review of biomarkers for disease progression in Alzheimer’s disease**


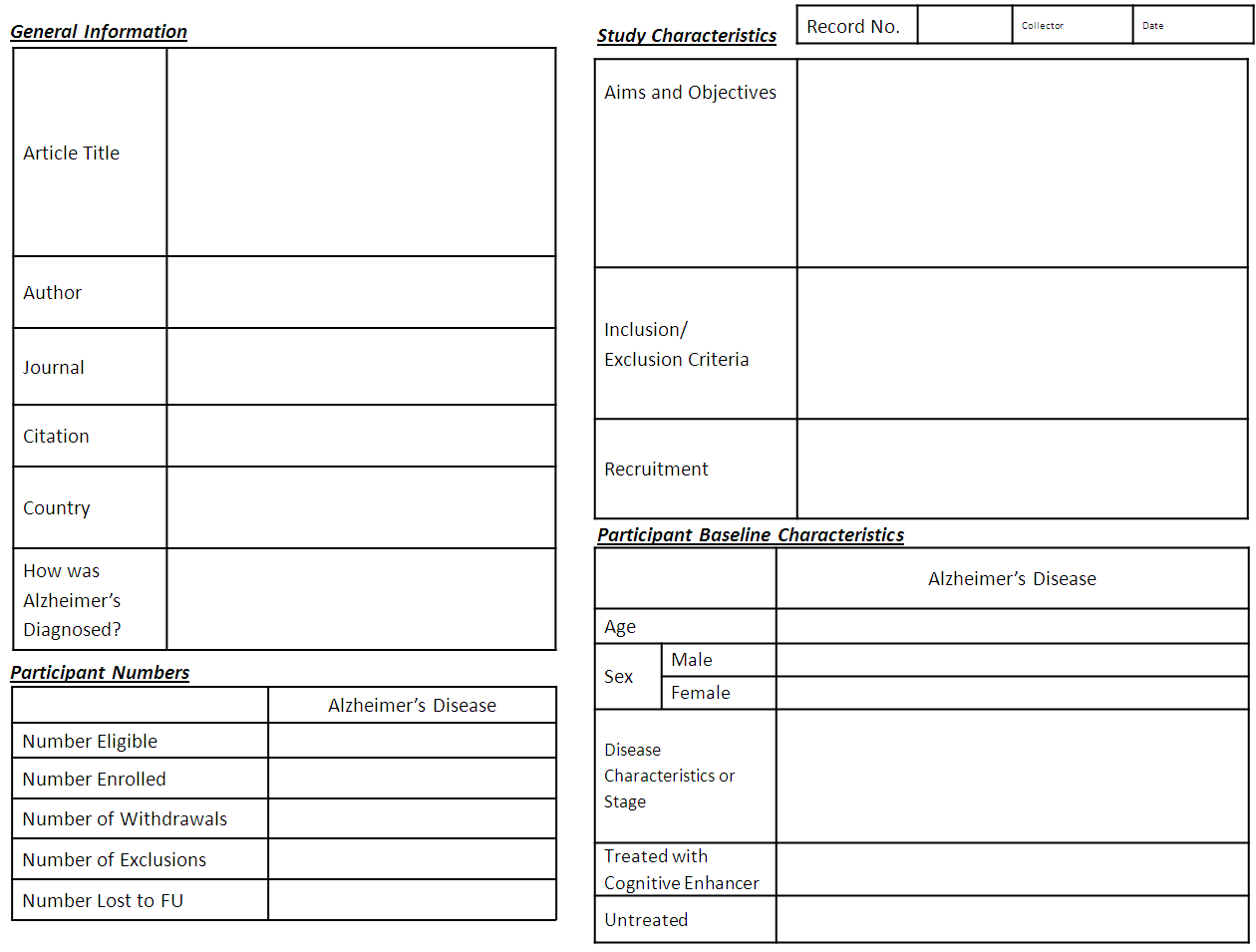


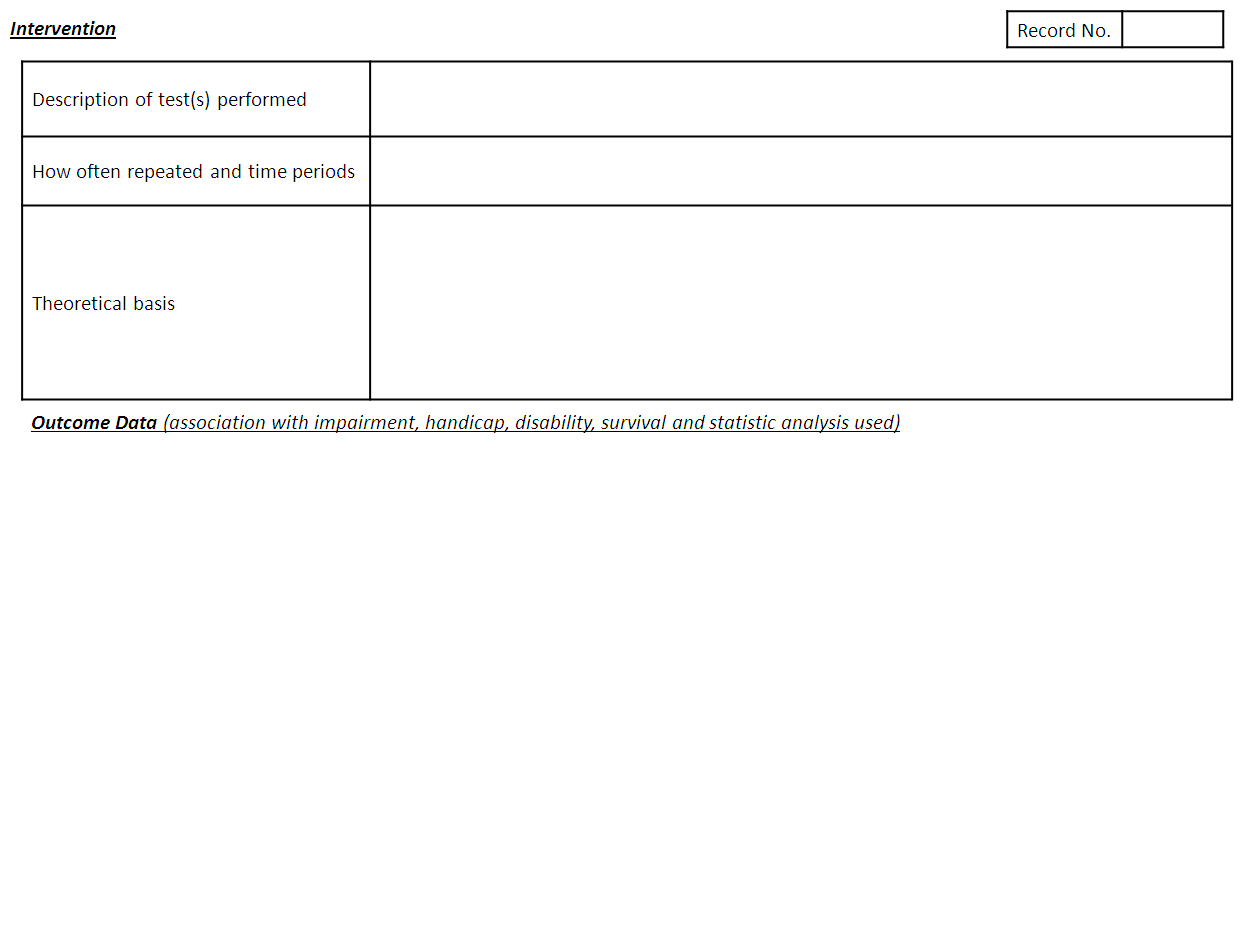


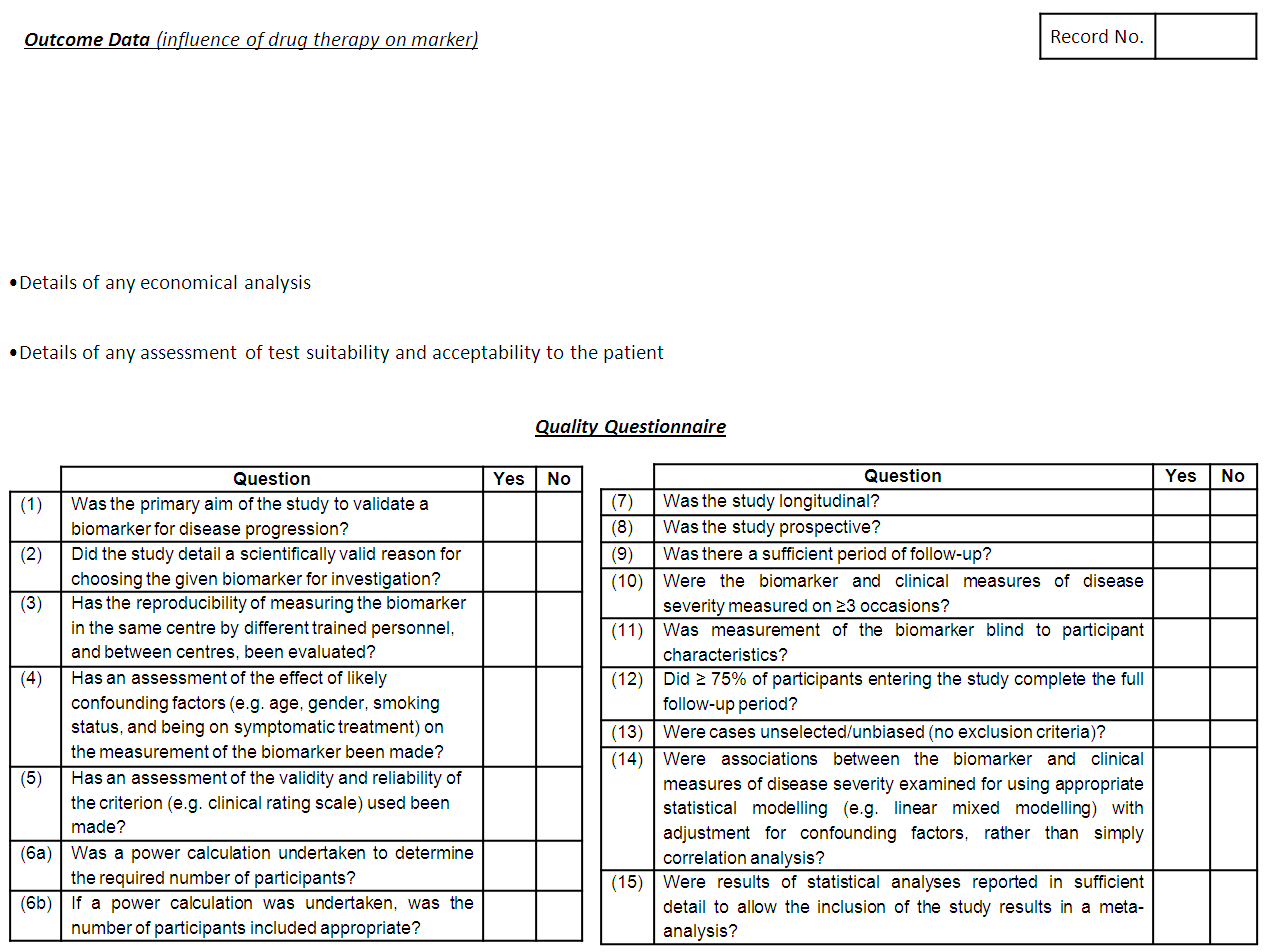

Supplement: Document S2 — Data extraction sheet. (DOCX) [file pone.0088854.s002.docx]
