# Supplementary material for: A Systematic Review of Biomarkers for Disease Progression in Alzheimer's Disease
Source: PLoS One. 2014 Feb 18;9(2):e88854. doi: 10.1371/journal.pone.0088854 (PMC3928315; doi:10.1371/journal.pone.0088854)
Supplement: Table S1 — Blood, plasma and serum biomarkers. (DOCX) [file pone.0088854.s003.docx]

# Table S1 *Blood/plasma/serum*

**Associations between putative blood, plasma and serum biomarkers and clinical measures of disease severity, in longitudinal studies included in the systemic review of biomarkers for disease progression in Alzheimer’s disease**

|  | | |  |  | **Association of change in substance measured with change in:** | | | | |
| --- | --- | --- | --- | --- | --- | --- | --- | --- | --- |
| **Substance measured** | **Reference**  **(first author, year)** | **n at baseline** | **Number of sampling intervals** | **Time between first and last samples (years)** | **MMSE** | **Modified ADAS-cog** | **CERAD battery** | **CIBIC+** | **DAD** |
| Platelet amyloid beta precursor protein (AβPP) isoform ratio | Baskin, 2000^1^ | 10 | 2 | 3.0 | R = 0.69(*) |  |  |  |  |
|  | Borroni, 2001^2^ | 20 | 2 | 0.1 | NSA |  |  |  |  |
|  | Liu, 2007^3^ | 66 | 2 | 1.0 | R = 0.316◘ |  |  |  |  |
| Neopterin | Blasko, 2007^4^ | 43 | 2 | 1.5 | R_s_ = -0.401**† |  | R_s_ = -0.353* |  |  |
| C-reactive protein (CRP) | Blasko, 2007^4^ | 43 | 2 | 1.5 | NSA† |  | NSA |  |  |
| Tumour necrosis factor-alpha (TNF-α) | Alvarez, 2009^5^ | 207 | 3 | 0.5 |  | NSA |  | NSA | NSA |
| Insulin-like growth factor-1  (IFG-1) | Alvarez, 2009^5^ | 207 | 3 | 0.5 |  | NSA |  | NSA | NSA |
|  | Alvarez, 2009^5^ | ? ‡ | 3 | 0.5 |  |  |  | R = -0.158◘ | R = 0.191* |
|  | Alvarez, 2009^5^ | ? § | 3 | 0.5 |  |  |  | R = -0.205◘ | R = 0.223* |
| Homeostasis model assessment (HOMA)• | Isik, 2009^6^ | 40 | 4 | 1.5 | NSA |  |  |  |  |
| Modified HOMA• | Isik, 2009^6^ | 40 | 4 | 1.5 | NSA |  |  |  |  |
| Afternoon cortisol test (ACT) | Weiner, 1997^7^ | 9 | 3 | 3.0 |  | POS* |  |  |  |
| 12:00 hours plasma cortisol concentration | Weiner, 1997^7^ | 9 | 3 | 3.0 |  | POS* |  |  |  |

**Key**

† Multiple regression analysis considering the influence of changes of neopterin and CRP on the CERAD battery (controlled for age, gender, ApoE4 presence and the time interval between both measurements) was also performed, in addition to the basic correlation analyses detailed in the table above. The change in z-scores relating to each of the subsections of the CERAD battery and the total sum of all seven CERAD z-scores were considered as dependent variables. The regression with variable MMSE, constructive praxis immediate recall and sum of all CERAD subscores were significantly associated with an increase of neopterin (P=0.009-0.036) and also with a decrease in CRP (P=0.008-0.037). The paper fails to state what the outcome of the regression was when the total CERAD battery score was the dependent variable.

‡ Exact number of patients included not clear, but does state examined a subgroup with late-onset Alzheimer’s disease.

§ Exact number of patients included not clear, but does state examined a subgroup of female patients with late-onset Alzheimer’s disease.

• The homeostasis model assessment (HOMA) is an estimate of steady state beta cell function and insulin sensitivity based on serum glucose and plasma insulin measurements.

Superscript numbers correspond to the list of references

**Correlations**

Pearson’s correlation coefficient R

Spearman’s correlation coefficient R_s_

NSA No significant association No symbol: P not significant, but actual value not stated

POS Significant positive association ◘ P ≥ 0.05

NEG Significant negative association ^(^*^)^ P significant, but actual value not stated

SIG Significant association direction not stated * P < 0.05

** P < 0.01

*** P < 0.001

**Clinical Rating Scales**

CERAD battery Consortium to Establish a Registry for Alzheimer’s Disease battery total score^8^

CIBIC+ Clinician Interview-Based Impression of Change with Caregiver Input (ADCS version)^9^

DAD Disability assessment for Dementia^10^

MMSE Mini-Mental State Examination^11^

Modified ADAS-cog Modified Alzheimer’s Disease Assessment Scale – cognitive subpart^12^ (modified as word recognition test omitted)

**References**

1. Baskin F, Rosenberg RN, Iyer L, Hynan L, Cullum CM (2000) Platelet APP isoform ratios correlate with declining cognition in AD. Neurology 54: 1907-1909.

2. Borroni B, Colciaghi F, Pastorino L, Pettenati C, Cottini E, et al. (2001) Amyloid precursor protein in platelets of patients with Alzheimer disease: effect of acetylcholinesterase inhibitor treatment. Arch Neurol 58: 442-446.

3. Liu HC, Wang HC, Ko SY, Wang PN, Chi CW, et al. (2007) Correlation between platelet amyloid precursor protein isoform ratio and cognition in Alzheimer's disease. J Alzheimers Dis 11: 77-84.

4. Blasko I, Knaus G, Weiss E, Kemmler G, Winkler C, et al. (2007) Cognitive deterioration in Alzheimer's disease is accompanied by increase of plasma neopterin. J Psychiatr Res 41: 694-701.

5. Alvarez XA, Sampedro C, Cacabelos R, Linares C, Aleixandre M, et al. (2009) Reduced TNF-alpha and increased IGF-I levels in the serum of Alzheimer's disease patients treated with the neurotrophic agent cerebrolysin. Int J Neuropsychopharmacol 12: 867-872.

6. Isik AT, Bozoglu E (2009) Acetylcholinesterase inhibition and insulin resistance in late onset Alzheimer's disease. Int Psychogeriatr 21: 1127-1133.

7. Weiner MF, Vobach S, Olsson K, Svetlik D, Risser RC (1997) Cortisol secretion and Alzheimer's disease progression. Biol Psychiatry 42: 1030-1038.

8. Welsh K, Butters N, Hughes J, Mohs R, Heyman A (1991) Detection of abnormal memory decline in mild cases of Alzheimer's disease using CERAD neuropsychological measures. Arch Neurol 48: 278-281.

9. Knopman DS, Knapp MJ, Gracon SI, Davis CS (1994) The Clinician Interview-Based Impression (CIBI): a clinician's global change rating scale in Alzheimer's disease. Neurology 44: 2315-2321.

10. Gelinas I, Gauthier L, McIntyre M, Gauthier S (1999) Development of a functional measure for persons with Alzheimer's disease: the disability assessment for dementia. Am J Occup Ther 53: 471-481.

11. Folstein MF, Folstein SE, McHugh PR (1975) "Mini-mental state". A practical method for grading the cognitive state of patients for the clinician. J Psychiatr Res 12: 189-198.

12. Mohs RC, Knopman D, Petersen RC, Ferris SH, Ernesto C, et al. (1997) Development of cognitive instruments for use in clinical trials of antidementia drugs: additions to the Alzheimer's Disease Assessment Scale that broaden its scope. The Alzheimer's Disease Cooperative Study. Alzheimer Dis Assoc Disord 11: S13-S21.
