# Supplementary material for: A Systematic Review of Biomarkers for Disease Progression in Alzheimer's Disease
Source: PLoS One. 2014 Feb 18;9(2):e88854. doi: 10.1371/journal.pone.0088854 (PMC3928315; doi:10.1371/journal.pone.0088854)
Supplement: Table S2 — CSF biomarkers. (DOCX) [file pone.0088854.s004.docx]

# Table S2 *Cerebrospinal fluid (CSF)*

**Associations between putative cerebrospinal fluid (CSF) biomarkers and clinical measures of disease severity, in longitudinal studies included in the systemic review of biomarkers for disease progression in Alzheimer’s disease**

|  | | |  |  | **Association of change in substance measured with change in:** | | | | | |
| --- | --- | --- | --- | --- | --- | --- | --- | --- | --- | --- |
| **Substance measured** | **Reference**  **(first author, year)** | **n at baseline** | **Number of sampling intervals** | **Time between first and last samples (years)** | **MMSE** | **ADAS-cog** | **CDR-SB** | **Blessed IMC** | **Blessed ADL** | **ADCS-ADL** |
| Total tau (T-tau) | Andreasen, 1999^1^ | 274† | 2 | 0.8 | R = -0.06 |  |  |  |  |  |
|  | Andreasen, 1998^2^ | 43 | 2 | 1.0 | R_s_ = 0.03◘ |  |  |  |  |  |
|  | Blennow, 2007^3^ | 65 | 2 | 0.5 | R = -0.08◘ | R = 0.11◘ |  |  |  |  |
|  | Buchhave, 2009^4^ | 100 | 2 | 1.2 | NSA◘ |  |  |  |  |  |
|  | Degerman, 2007^5^ | 11 | 2 | 1.0 |  |  |  |  |  | NSA |
|  | Hampel, 2001^6^ | 17‡ | ? ‡ | ? ‡ | NSA |  |  |  |  |  |
|  | Tapiola, 2000^7^ | 17 | 2 | 3.1 | NSA |  |  |  |  |  |
|  | Vemuri, 2010^8^ | 71 | 2 | 1.0 | R_s_ = 0.06◘ | R_s_ = 0.14◘ | R_s_ = -0.03◘ |  |  |  |
| Tau phosphorylated at threonine 181 (p-tau_181_) | Blennow, 2007^3^ | 65 | 2 | 0.5 | R = 0.04◘ | R = 0.11◘ |  |  |  |  |
|  | Degerman, 2007^5^ | 11 | 2 | 1.0 |  |  |  |  |  | NSA |
|  | Seppälä, 2011^9^ | 56 | 2 | 3.1 | R_s_ = 0.579*** |  |  |  |  |  |
|  | Seppälä, 2011^9^ | 15§ | 2 | 3.1 | R_s_ = 0.617* |  |  |  |  |  |
| Tau phosphorylated at threonine 231 (p-tau_231_) | Hampel, 2001^6^ | 17‡ | ? ‡ | ? ‡ | NSA |  |  |  |  |  |
| 42-amino acid isoform of amyloid beta (Aβ42) | Blennow, 2007^3^ | 65 | 2 | 0.5 | R = -0.08◘ | R = 0.04◘ |  |  |  |  |
|  | Buchhave, 2009^4^ | 100 | 2 | 1.2 | NSA◘ |  |  |  |  |  |
|  | Degerman, 2007^5^ | 11 | 2 | 1.0 |  |  |  |  |  | NSA |
|  | Höglund, 2005^10^ | 18 | 2 | 1.0 | NSA | NSA |  |  |  |  |
|  | Tapiola, 2000^7^ | 17 | 2 | 3.1 | NSA |  |  |  |  |  |
|  | Vemuri, 2010^8^ | 71 | 2 | 1.0 | R_s_ = 0.30* | R_s_ = 0.11◘ | R_s_ = -0.11◘ |  |  |  |

|  | | |  |  | **Association of change in substance measured with change in:** | | | | | |
| --- | --- | --- | --- | --- | --- | --- | --- | --- | --- | --- |
| **Substance measured** | **Reference**  **(first author, year)** | **n at baseline** | **Number of sampling intervals** | **Time between first and last samples (years)** | **MMSE** | **ADAS-cog** | **CDR-SB** | **Blessed IMC** | **Blessed ADL** | **ADCS-ADL** |
| 38-amino acid isoform of amyloid beta (Aβ38) | Höglund, 2005^10^ | 18 | 2 | 1.0 | SIG* | NSA |  |  |  |  |
| 37-amino acid isoform of amyloid beta (Aβ37) | Höglund, 2005^10^ | 18 | 2 | 1.0 | NSA | NSA |  |  |  |  |
| Soluble amyloid beta (sAβ) | Pirttilä, 1998^11^ | 25 | 2 | 3.0 | R = 0.77*** |  |  |  |  |  |
| Acetylcholinesterase (AChE) activity | Elble, 1987^12^ | 10 | 3 | 1.0 | NSA◘ |  |  | NSA◘ | NSA◘ |  |

**Key**

† A second lumbar puncture was only performed in 150 patients with probable Alzheimer’s disease.

‡ This paper stated that up to seven spinal taps, mean interval 17.0 (SD 14.8) months, were performed on 17 patients with probable Alzheimer’s disease. The change in MMSE was calculated and, therefore, it would appear to have been recorded more than once.

§ Subgroup analysis of 15 patients with a definite neuropathological diagnosis of Alzheimer’s disease. The rest of the cohort of 56 patients had only a clinical diagnosis of probable Alzheimer’s disease

Superscript numbers correspond to the list of references

**Correlations**

Pearson’s correlation coefficient R

Spearman’s correlation coefficient R_s_

NSA No significant association No symbol: P not significant, but actual value not stated

POS Significant positive association ◘ P ≥ 0.05

NEG Significant negative association ^(^*^)^ P significant, but actual value not stated

SIG Significant association direction not stated * P < 0.05

** P < 0.01

*** P < 0.001

**Clinical Rating Scales**

ADAS-cog Alzheimer’s Disease Assessment Scale – cognitive subscale^13^

ADCS-ADL Alzheimer’s Disease Cooperative Study Activities of Daily Living inventory^14^

Blessed ADL Blessed Dementia Scale change in performance of everyday activities subsection^15^

Blessed IMC Blessed Dementia Information-Memory-Concentration test^15^

CDR-SB The Washington University Clinical Dementia Rating Sum-of-Boxes score^16^

MMSE Mini-Mental State Examination^17^

**References**

1. Andreasen N, Minthon L, Clarberg A, Davidsson P, Gottfries J, et al. (1999) Sensitivity, specificity, and stability of CSF-tau in AD in a community- based patient sample. Neurology 53: 1488-1494.

2. Andreasen N, Vanmechelen E, Van de Voorde A, Davidsson P, Hesse C, et al. (1998) Cerebrospinal fluid tau protein as a biochemical marker for Alzheimer's disease: A community based follow up study. J Neurol Neurosurg Psychiatry 64: 298-305.

3. Blennow K, Zetterberg H, Minthon L, Lannfelt L, Strid S, et al. (2007) Longitudinal stability of CSF biomarkers in Alzheimer's disease. Neurosci Lett 419: 18-22.

4. Buchhave P, Blennow K, Zetterberg H, Stomrud E, Londos E, et al. (2009) Longitudinal study of CSF biomarkers in patients with Alzheimer's disease. PLoS One 4: e6294. 10.1371/journal.pone.0006294 [doi].

5. Degerman GM, Kilander L, Basun H, Lannfelt L (2007) Reduction of phosphorylated tau during memantine treatment of Alzheimer's disease. Dement Geriatr Cogn Disord 24: 247-252.

6. Hampel H, Buerger K, Kohnken R, Teipel SJ, Zinkowski R, et al. (2001) Tracking of Alzheimer's disease progression with cerebrospinal fluid tau protein phosphorylated at threonine 231. Ann Neurol 49: 545-546.

7. Tapiola T, Pirttila T, Mikkonen M, Mehta PD, Alafuzoff I, et al. (2000) Three-year follow-up of cerebrospinal fluid tau, beta-amyloid 42 and 40 concentrations in Alzheimer's disease. Neurosci Lett 280: 119-122.

8. Vemuri P, Wiste HJ, Weigand SD, Knopman DS, Trojanowski JQ, et al., Alzheimer's Disease Neuroimaging Initiative (2010) Serial MRI and CSF biomarkers in normal aging, MCI, and AD. Neurology 75: 143-151.

9. Seppala TT, Koivisto AM, Hartikainen P, Helisalmi S, Soininen H, et al. (2011) Longitudinal changes of CSF biomarkers in alzheimer's disease. J Alzheimers Dis 25: 583-594.

10. Hoglund K, Syversen S, Lewczuk P, Wallin A, Wiltfang J, et al. (2005) Statin treatment and a disease-specific pattern of beta-amyloid peptides in Alzheimer's disease. Exp Brain Res 164: 205-214.

11. Pirttila T, Koivisto K, Mehta PD, Reinikainen K, Kim KS, et al. (1998) Longitudinal study of cerebrospinal fluid amyloid proteins and apolipoprotein E in patients with probable Alzheimer's disease. Neurosci Lett 249: 21-24.

12. Elble R, Giacobini E, Scarsella GF (1987) Cholinesterases in cerebrospinal fluid. A longitudinal study in Alzheimer disease. Arch Neurol 44: 403-407.

13. Mohs RC, Knopman D, Petersen RC, Ferris SH, Ernesto C, et al. (1997) Development of cognitive instruments for use in clinical trials of antidementia drugs: additions to the Alzheimer's Disease Assessment Scale that broaden its scope. The Alzheimer's Disease Cooperative Study. Alzheimer Dis Assoc Disord 11: S13-S21.

14. Galasko D, Bennett D, Sano M, Ernesto C, Thomas R, et al. (1997) An inventory to assess activities of daily living for clinical trials in Alzheimer's disease. The Alzheimer's Disease Cooperative Study. Alzheimer Dis Assoc Disord 11: S33-S39.

15. Blessed G, Tomlinson BE, Roth M (1968) The association between quantitative measures of dementia and of senile change in the cerebral grey matter of elderly subjects. Br J Psychiatry 114: 797-811.

16. Morris JC (1993) The Clinical Dementia Rating (CDR): current version and scoring rules. Neurology 43: 2412-2414.

17. Folstein MF, Folstein SE, McHugh PR (1975) "Mini-mental state". A practical method for grading the cognitive state of patients for the clinician. J Psychiatr Res 12: 189-198.
