# Supplementary material for: A Systematic Review of Biomarkers for Disease Progression in Alzheimer's Disease
Source: PLoS One. 2014 Feb 18;9(2):e88854. doi: 10.1371/journal.pone.0088854 (PMC3928315; doi:10.1371/journal.pone.0088854)
Supplement: Table S3 — Ultrasound biomarkers. (DOCX) [file pone.0088854.s005.docx]

# Table S3 *Ultrasound*

**Associations between putative ultrasound biomarkers and clinical measures of disease severity, in longitudinal studies included in the systemic review of biomarkers for disease progression in Alzheimer’s disease**

|  | | | |  |  | **Association of change in feature measured with change in:** | | |
| --- | --- | --- | --- | --- | --- | --- | --- | --- |
| **Modality** | **Feature measured** | **Reference**  **(first author, year)** | **n at baseline** | **Number of scans** | **Time between first and last scan (years)** | **MMSE** | **Total CAMCOG** | **CAMCOG (memory)** |
| Colour-coded sonography | Intima-media thickness (ITT) of common carotid arteries  (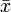) | Silvestrini, 2009^1^ | 66 | 2 | 1.0 | β = 5.11***† |  |  |
|  | Plaque index (PI)  [sum of right and left plaque indices] | Silvestrini, 2009^1^ | 66 | 2 | 1.0 | β = 1.60**† |  |  |

**Key**

† Multiple linear regression was used to determine whether the severity of cognitive decline, as measured by MMSE, was related to atherosclerotic ultrasound markers. Before running the main regression adjustments were made: (1) IMT changes adjusted for baseline IMT; (2) PI worsening adjusted for baseline IMT; (3) MMSE change was adjusted for baseline MMSE and age. Subsequently multiple regression was undertaken with adjusted MMSE change over one year as the dependent variable and adjusted IMT change, adjusted PI change, baseline IMT, baseline MMSE, hypertension and age as independent variables. The final overall model produced was: MMSE score decrease (adjusted) = -1.05 + 1.91(baseline IMT) + 5.11(IMT-adjusted change) + 1.60(PI-adjusted change) – 1.78(1 if on antihypertensive; 0 otherwise).


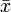
 Where this symbol is show then the value given is the average of left and right structures. If not shown then it is unclear from the text whether the value represents an average or a total (left and right hemispheric structures combined) value.

Superscript numbers correspond to the list of references

**Correlations**

Beta coefficient β

NSA No significant association No symbol: P not significant, but actual value not stated

POS Significant positive association ◘ P ≥ 0.05

NEG Significant negative association ^(^*^)^ P significant, but actual value not stated

SIG Significant association direction not stated * P < 0.05

** P < 0.01

*** P < 0.001

**Clinical Rating Scales**

CAMCOG (memory) The memory subsection of the Cambridge Examination for Mental disorders of the Elderly^2^

MMSE Mini-Mental State Examination^3^

Total CAMCOG The cognitive and self-contained part of the Cambridge Examination for Mental disorders of the Elderly^2^

**References**

1. Silvestrini M, Gobbi B, Pasqualetti P, Bartolini M, Baruffaldi R, et al. (2009) Carotid atherosclerosis and cognitive decline in patients with Alzheimer's disease. Neurobiol Aging 30: 1177-1183.

2. Roth M, Tym E, Mountjoy CQ, Huppert FA, Hendrie H, et al. (1986) CAMDEX. A standardised instrument for the diagnosis of mental disorder in the elderly with special reference to the early detection of dementia. Br J Psychiatry 149: 698-709.

3. Folstein MF, Folstein SE, McHugh PR (1975) "Mini-mental state". A practical method for grading the cognitive state of patients for the clinician. J Psychiatr Res 12: 189-198.
