# Supplementary material for: A Systematic Review of Biomarkers for Disease Progression in Alzheimer's Disease
Source: PLoS One. 2014 Feb 18;9(2):e88854. doi: 10.1371/journal.pone.0088854 (PMC3928315; doi:10.1371/journal.pone.0088854)
Supplement: Table S4 — CT brain biomarkers. (DOCX) [file pone.0088854.s006.docx]

# Table S4 *CT brain*

**Associations between putative CT brain biomarkers and clinical measures of disease severity, in longitudinal studies included in the systemic review of biomarkers for disease progression in Alzheimer’s disease**

|  | | | |  |  | **Association of change in feature measured with change in:** | | |
| --- | --- | --- | --- | --- | --- | --- | --- | --- |
| **Modality** | **Feature measured** | **Reference**  **(first author, year)** | **n at baseline** | **Number of scans** | **Time between first and last scan (years)** | **MMSE** | **Total CAMCOG** | **CAMCOG (memory)** |
| Non-contrast CT | Ventricular size | Burns, 1991^1^ | 63 | 2 | 1.0 |  | R = -0.21* | R = -0.24* |

**Key**


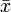
 Where this symbol is show then the value given is the average of left and right hemispheric structures. If not shown then it is unclear from the text whether the value represents an average or a total (left and right hemispheric structures combined) value.

Superscript numbers correspond to the list of references

**Correlations**

Pearson’s correlation coefficient R

NSA No significant association No symbol: P not significant, but actual value not stated

POS Significant positive association ◘ P ≥ 0.05

NEG Significant negative association ^(^*^)^ P significant, but actual value not stated

SIG Significant association direction not stated * P < 0.05

** P < 0.01

*** P < 0.001

**Clinical Rating Scales**

CAMCOG (memory) The memory subsection of the Cambridge Examination for Mental disorders of the Elderly^2^

MMSE Mini-Mental State Examination^3^

Total CAMCOG The cognitive and self-contained part of the Cambridge Examination for Mental disorders of the Elderly^2^

**References**

1. Burns A, Jacoby R, Levy R (1991) Computed tomography in Alzheimer's disease: a longitudinal study. Biol Psychiatry 29: 383-390.

2. Roth M, Tym E, Mountjoy CQ, Huppert FA, Hendrie H, et al. (1986) CAMDEX. A standardised instrument for the diagnosis of mental disorder in the elderly with special reference to the early detection of dementia. Br J Psychiatry 149: 698-709.

3. Folstein MF, Folstein SE, McHugh PR (1975) "Mini-mental state". A practical method for grading the cognitive state of patients for the clinician. J Psychiatr Res 12: 189-198.
