# Supplementary material for: A Systematic Review of Biomarkers for Disease Progression in Alzheimer's Disease
Source: PLoS One. 2014 Feb 18;9(2):e88854. doi: 10.1371/journal.pone.0088854 (PMC3928315; doi:10.1371/journal.pone.0088854)
Supplement: Table S5 — Brain MRI biomarkers. (DOCX) [file pone.0088854.s007.docx]

# Table S5 *Brain MRI*

**Associations between putative brain MRI biomarkers and clinical measures of disease severity, in longitudinal studies included in the systemic review of biomarkers for disease progression in Alzheimer’s disease**

|  | | | |  |  | **Association of change in feature measured with change in:** | | | | | | | |
| --- | --- | --- | --- | --- | --- | --- | --- | --- | --- | --- | --- | --- | --- |
| **Modality** | **Feature measured** | **Reference**  **(first author, year)** | **n at BL** | **Number of scans** | **Time between first and last scan (years)** | **MMSE** | **ADAS-cog** | **Global CDR** | **CDR-SB** | **DRS** | **BCRS** | **DAD** | **FAQ** |
| T1-weighted volumetric MRI | Whole brain volume | Evans, 2010^1^ | 99 | 2 | 1.0 | POS** | NEG◘ |  |  |  |  |  |  |
|  |  | Fox, 1999^2^ | 2 | ≥2 | ? † | R = 0.80*** |  |  |  |  |  |  |  |
|  |  | Hua, 2010^3^ | 144 | 2 | 1 | NSA | NSA |  | NSA |  |  |  | NSA |
|  |  | Jack, 2004^4^ | 64 | 2 | 1.3 | R_s_ = 0.47*** |  |  | R_s_ = -0.45*** | R_s_ = 0.47*** |  |  |  |
|  |  | Joseph, 2008^5^ | 32 | 2 | 1.4 | R_p_ = 0.23◘ |  |  | R_p_ = -0.28◘ |  |  |  |  |
|  |  | Pantel, 2002^6^ | 13 | 2 | ‡ | R = 0.44 |  |  |  |  | R = -0.11 |  |  |
|  |  | Ridha, 2008^7^ | 52 | 2 | 0.9 | R = 0.59*** | R = -0.35* | R = - 0.28* |  |  |  | R = 0.43** |  |
|  |  | Schott, 2008^8^ | 46 | 9§ | 2.0 | NSA◘ |  |  |  |  |  |  |  |
|  |  | Sluimer, 2008^9^ | 65 | 2 | 1.7 | R_p_ = 0.34** |  |  |  |  |  |  |  |
|  | Temporal lobe volume | Hua, 2010^3^ | 144 | 2 | 1 | NSA | NEG*** |  | NEG*** |  |  |  | NSA |
|  |  | Leow, 2009^10^ | 20 | 2 | 1 | POS◘ |  | NEG◘ |  |  |  |  |  |
|  | Entorhinal cortex volume | Jack, 2004^4^ | 64 | 2 | 1.3 | R_s_ = 0.46*** |  |  | R_s_ = -0.12◘ | R_s_ = 0.21◘ |  |  |  |

|  | | | |  |  | **Association of change in feature measured with change in:** | | | | | | |
| --- | --- | --- | --- | --- | --- | --- | --- | --- | --- | --- | --- | --- |
| **Modality** | **Feature measured** | **Reference**  **(first author, year)** | **n at BL** | **Number of scans** | **Time between first and last scan (years)** | **MMSE** | **ADAS-cog** | **Global CDR** | **CDR-SB** | **DRS** | **BCRS** | **DAD** |
| T1-weighted volumetric MRI  (cont.) | Hippocampal volume | Gauthier, 2009^11^ | 312 | 2 | 1.5 |  | R_?_ = -0.08◘ |  | R_?_ = -0.04◘ |  |  |  |
|  |  | Jack, 2004^4^ | 64 | 2 | 1.3 | R_s_ = 0.35* |  |  | R_s_ = -0.24◘ | R_s_ = 0.16◘ |  |  |
|  |  | Morra, 2009^12^ | 97 | 2 | 1.0 | R = 0.039◘ |  | R = -0.193* | R = -0.093◘ |  |  |  |
|  |  | Morra, 2009^12^ | 97 | 2 | 1.0 | R = 0.098◘ |  | R = -0.104◘ | R = -0.150◘ |  |  |  |
|  |  | Pantel, 2002^6^ | 13 | 2 | ‡ | R = 0.6* |  |  |  |  | R = -0.66* |  |
|  |  | Ridha, 2008^7^ | 52 | 2 | 0.9 | R = 0.17◘ | R = -0.02◘ | R = -0.04◘ |  |  |  | R = 0.03◘ |
|  |  | Thompson, 2004^13^ | 12 | 2 | 1.5 | NSA |  |  |  |  |  |  |
|  | Temporal horn volume | Thompson, 2004^13^ | 12 | 2 | 1.5 | NSA |  |  |  |  |  |  |
|  | Inferior ventricular horn volume |  |  |  |  |  |  |  |  |  |  |  |
|  | Left | Thompson, 2004^13^ | 12 | 2 | 1.5 | NEG* |  |  |  |  |  |  |
|  | Right | Thompson, 2004^13^ | 12 | 2 | 1.5 | NEG* |  |  |  |  |  |  |

|  | | | |  |  | **Association of change in feature measured with change in:** | | | | | |
| --- | --- | --- | --- | --- | --- | --- | --- | --- | --- | --- | --- |
| **Modality** | **Feature measured** | **Reference**  **(first author, year)** | **n at BL** | **Number of scans** | **Time between first and last scan (years)** | **MMSE** | **ADAS-cog** | **Global CDR** | **CDR-SB** | **DRS** | **DAD** |
| T1-weighted volumetric MRI  (cont.) | Ventricular volume | Evans, 2010^1^ | 99 | 2 | 1.0 | NEG** | POS** |  |  |  |  |
|  |  | Jack, 2004^4^ | 64 | 2 | 1.3 | R_s_ = -0.38*** |  |  | R_s_ = 0.40*** | R_s_ = -0.39* |  |
|  |  | Joseph, 2008^5^ | 32 | 2 | 1.4 | R_p_ = -0.72*** |  |  | R_p_ = 0.37◘ |  |  |
|  |  | Kantarci, 2007^14^ | 60 | 2 | 1.1 | R_s_ = -0.32* |  |  | R_s_ = 0.39** | R_s_ = -0.34* |  |
|  |  | Nestor, 2008^15^ | 18 | 2 | 0.5 |  | R = 0.627** |  |  |  |  |
|  |  | Ridha, 2008^7^ | 52 | 2 | 0.9 | R = -0.50*** | R = 0.40** | R = 0.25◘ |  |  | R = -0.56*** |
|  |  | Vemuri, 2010^16^ | 71 | 2 | 1.0 | R_s_ = -0.31* | R_s_ = 0.32* |  | Rs = 0.38*** |  |  |
|  | Compound measure• |  |  |  |  |  |  |  |  |  |  |
|  | Left | Thompson, 2004^13^ | 12 | 2 | 1.5 | NEG*** |  |  |  |  |  |
|  | Right | Thompson, 2004^13^ | 12 | 2 | 1.5 | NEG* |  |  |  |  |  |
|  | Total area of corpus callosum | Teipel, 2002^17^ | 27 | 4 ^Φ^ | 4.0 | NSA¥ |  |  |  |  |  |
|  | Area of collosal rostrum and genu (C1) | Teipel, 2002^17^ | 27 | 4 ^Φ^ | 4.0 | NSA¥ |  |  |  |  |  |
|  | Area of anterior truncus (C2) | Teipel, 2002^17^ | 27 | 4 ^Φ^ | 4.0 | NSA¥ |  |  |  |  |  |
|  | Area of middle truncus (C3) | Teipel, 2002^17^ | 27 | 4 ^Φ^ | 4.0 | NSA¥ |  |  |  |  |  |
|  | Area of posterior truncus (C4) | Teipel, 2002^17^ | 27 | 4 ^Φ^ | 4.0 | NSA¥ |  |  |  |  |  |
|  | Area of callosal isthmus and splenium (C5) | Teipel, 2002^17^ | 27 | 4 ^Φ^ | 4.0 | β = 0.446*¥ |  |  |  |  |  |

**Key**

BL Baseline

† This study simply stated that patients underwent two or more MRI scans with corresponding MMSE measurements. The mean interval of observations was 1.8 (SD 1.4) years.

‡ This paper only stated that patients with Alzheimer’s disease were seen ‘at intervals ranging from 12-38 months’ and that ‘volumetric MRI was performed in all subjects at both clinical examinations’.

§ In this study there were seven MRI scans (baseline, two, six, 12, 26, 38 and 52 weeks). In addition, 26 patients were also scanned at 18 months, and 14 of those were also scanned again at two years. However, cognitive testing was only performed at baseline and one year. Associations were then drawn between the annualized score reduction in MMSE and whole brain atrophy rate, in percent per year, using linear mixed modelling.

• Compound measure comprised ventricular gain rate, in percent per year, plus hippocampal loss rate, in percent per year.

^Φ^ In this study of the 27 patients included at baseline only 21 were scanned longitudinally (twice, n=14; three times, n=6; four times n=1).

¥ Stepwise linear multiple regression model was used to predict the individual percentage rates of change in each corpus callosum area on the basis of age at baseline, gender, white matter load, length of observation time, and annual rate of point loss in MMSE score. In the first step all variables were forced into the equation to assess the amount of variance explained by the selected model. Variables were then stepwise removed from the model when the amount of explained variance, which was contributed to the model, was below a threshold of F=2.71 (corresponding to P=0.10). Annual point loss in MMSE score was correlated significantly with percent reduction of corpus callosum in area C5, both within the full regression model (β = 0.446, P < 0.03), controlling for age, gender, observation time and white matter load, and in the single-effect analysis (R = 0.52, P < 0.02). Note: for other corpus callosum areas the paper simply stated that there no relationship between area reduction and point loss in MMSE score.


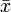
 Where this symbol is show then the value given is the average of left and right hemispheric structures. If not shown then it is unclear from the text whether the value represents an average or a total (left and right hemispheric structures combined) value.

Note: to allow comparisons to be made been papers the direction of associations were adjusted. The directions shown reflect those of an association drawn between a change in a clinical measure (clinical measure at time point 2 minus clinical measure at baseline) with a change in given structure on MRI (e.g. volume at time point 2 minus volume at baseline).

Superscript numbers correspond to the list of references

**Correlations**

Pearson’s correlation coefficient R

Spearman’s correlation coefficient R_s_

Partial correlation coefficient R_p_

Correlation coefficient unspecified R_?_

Beta coefficient β

NSA No significant association No symbol: P not significant, but actual value not stated

POS Significant positive association ◘ P ≥ 0.05

NEG Significant negative association ^(^*^)^ P significant, but actual value not stated

SIG Significant association direction not stated * P < 0.05

** P < 0.01

*** P < 0.001

**Clinical Rating Scales**

ADAS-cog Alzheimer’s Disease Assessment Scale – cognitive subscale^18^

BCRS Brief cognitive rating scale^19^

CDR-SB The Washington University Clinical Dementia Rating Sum-of-Boxes score^20^

DAD Disability assessment for Dementia^21^

DRS Dementia Rating Scale^22^

FAQ Functional Assessment Questionnaire^23^

Global CDR The Washington University Clinical Dementia Rating global score^20^

MMSE Mini-Mental State Examination^24^

**References**

1. Evans MC, Barnes J, Nielsen C, Kim LG, Clegg SL, et al. (2010) Volume changes in Alzheimer's disease and mild cognitive impairment: cognitive associations. Eur Radiol 20: 674-682.

2. Fox NC, Scahill RI, Crum WR, Rossor MN (1999) Correlation between rates of brain atrophy and cognitive decline in AD. Neurology 52: 1687-1689.

3. Hua X, Hibar DP, Lee S, Toga AW, Jack CR, et al. (2010) Sex and age differences in atrophic rates: An ADNI study with n=1368 MRI scans. Neurobiol Aging 31: 1463-1480.

4. Jack J, Shiung MM, Gunter JL, O'Brien PC, Weigand SD, et al. (2004) Comparison of different MRI brain athrophy rate measures with clinical disease progression in AD. Neurology 62: 591-600.

5. Josephs KA, Whitwell JL, Ahmed Z, Shiung MM, Weigand SD, et al. (2008) Beta-amyloid burden is not associated with rates of brain atrophy. Ann Neurol 63: 204-212.

6. Pantel J, Schonknecht P, Essig M, Amann M, Eysenbach K, et al. (2002) Progressive medial temporal lobe changes in Alzheimer's disease revealed by quantitative MRI: Potential use for monitoring of drug-related changes. Drug Dev Res 56: 51-56.

7. Ridha BH, Anderson VM, Barnes J, Boyes RG, Price SL, et al. (2008) Volumetric MRI and cognitive measures in Alzheimer disease: Comparison of markers of progression. J Neurol 255: 567-574.

8. Schott JM, Crutch SJ, Frost C, Warrington EK, Rossor MN, et al. (2008) Neuropsychological correlates of whole brain atrophy in Alzheimer's disease. Neuropsychologia 46: 1732-1737.

9. Sluimer JD, van der Flier WM, Karas GB, Fox NC, Scheltens P, et al. (2008) Whole-brain atrophy rate and cognitive decline: longitudinal MR study of memory clinic patients. Radiology 248: 590-598.

10. Leow AD, Yanovsky I, Parikshak N, Hua X, Lee S, et al. (2009) Alzheimer's Disease Neuroimaging Initiative: A one-year follow up study using tensor-based morphometry correlating degenerative rates, biomarkers and cognition. Neuroimage 45: 645-655.

11. Gauthier S, Aisen PS, Ferris SH, Saumier D, Duong A, et al. (2009) Effect of tramiprosate in patients with mild-to-moderate Alzheimer's disease: exploratory analyses of the MRI sub-group of the Alphase study. Journal of Nutrition, Health & Aging 13: 550-557.

12. Morra JH, Tu Z, Apostolova LG, Green AE, Avedissian C, et al. (2009) Automated mapping of hippocampal atrophy in 1-year repeat MRI data from 490 subjects with Alzheimer's disease, mild cognitive impairment, and elderly controls. Neuroimage 45: S3-S15.

13. Thompson PM, Hayashi KM, De Zubicaray GI, Janke AL, Rose SE, et al. (2004) Mapping hippocampal and ventricular change in Alzheimer disease. Neuroimage 22: 1754-1766.

14. Kantarci K, Weigand SD, Petersen RC, Boeve BF, Knopman DS, et al. (2007) Longitudinal 1H MRS changes in mild cognitive impairment and Alzheimer's disease. Neurobiol Aging 28: 1330-1339.

15. Nestor SM, Rupsingh R, Borrie M, Smith M, Accomazzi V, et al. (2008) Ventricular enlargement as a possible measure of Alzheimer's disease progression validated using the Alzheimer's disease neuroimaging initiative database. Brain 131: 2443-2454.

16. Vemuri P, Wiste HJ, Weigand SD, Knopman DS, Trojanowski JQ, et al., Alzheimer's Disease Neuroimaging Initiative (2010) Serial MRI and CSF biomarkers in normal aging, MCI, and AD. Neurology 75: 143-151.

17. Teipel SJ, Bayer W, Alexander GE, Zebuhr Y, Teichberg D, et al. (2002) Progression of corpus callosum atrophy in Alzheimer disease. Arch Neurol 59: 243-248.

18. Mohs RC, Knopman D, Petersen RC, Ferris SH, Ernesto C, et al. (1997) Development of cognitive instruments for use in clinical trials of antidementia drugs: additions to the Alzheimer's Disease Assessment Scale that broaden its scope. The Alzheimer's Disease Cooperative Study. Alzheimer Dis Assoc Disord 11: S13-S21.

19. Reisberg B, Ferris SH (1988) Brief Cognitive Rating Scale (BCRS). Psychopharmacol Bull 24: 629-636.

20. Morris JC (1993) The Clinical Dementia Rating (CDR): current version and scoring rules. Neurology 43: 2412-2414.

21. Gelinas I, Gauthier L, McIntyre M, Gauthier S (1999) Development of a functional measure for persons with Alzheimer's disease: the disability assessment for dementia. Am J Occup Ther 53: 471-481.

22. Brown GG, Rahill AA, Gorell JM, McDonald C, Brown SJ, et al. (1999) Validity of the Dementia Rating Scale in assessing cognitive function in Parkinson's disease. J Geriatr Psychiatry Neurol 12: 180-188.

23. Pfeffer RI, Kurosaki TT, Harrah CH, Jr., Chance JM, Filos S (1982) Measurement of functional activities in older adults in the community. J Gerontol 37: 323-329.

24. Folstein MF, Folstein SE, McHugh PR (1975) "Mini-mental state". A practical method for grading the cognitive state of patients for the clinician. J Psychiatr Res 12: 189-198.
