# Supplementary material for: A Systematic Review of Biomarkers for Disease Progression in Alzheimer's Disease
Source: PLoS One. 2014 Feb 18;9(2):e88854. doi: 10.1371/journal.pone.0088854 (PMC3928315; doi:10.1371/journal.pone.0088854)
Supplement: Table S6 — Brain MRS biomarkers. (DOCX) [file pone.0088854.s008.docx]

# Table S6 *Brain MRS*

**Associations between putative brain MRS biomarkers and clinical measures of disease severity, in longitudinal studies included in the systemic review of biomarkers for disease progression in Alzheimer’s disease**

|  | | | |  |  | **Association of change in feature measured with change in:** | | | | | |
| --- | --- | --- | --- | --- | --- | --- | --- | --- | --- | --- | --- |
| **Region of brain where feature measured** | **Feature measured** | **Reference**  **(first author, year)** | **n at baseline** | **Number of scans** | **Time between first and last scan (years)** | **MMSE** | **ADAS-cog** | **ADAS-noncog** | **CDR-SB** | **DRS** | **IDDD** |
| Bilateral anterior cingulate cortex | NAA/Cr ratio | Modrego, 2006^1^ | 24 | 2 | 0.3 |  | R = 0.54** | NSA |  |  | R = 0.54** |
|  | mI/Cr ratio | Modrego, 2006^1^ | 24 | 2 | 0.3 |  | NSA | R = 0.51* |  |  | R = 0.49* |
| Pre-frontal region |  |  |  |  |  |  |  |  |  |  |  |
| Right | NAA/Cr ratio | Modrego, 2010^2^ | 63 | 2 | 0.5 |  | NSA |  |  |  |  |
| Left | NAA/Cr ratio | Modrego, 2010^2^ | 63 | 2 | 0.5 |  | NSA |  |  |  |  |
| Temporal lobe |  |  |  |  |  |  |  |  |  |  |  |
| Right | NAA/Cr ratio | Modrego, 2010^2^ | 63 | 2 | 0.5 |  | NSA |  |  |  |  |
| Left | NAA/Cr ratio | Modrego, 2010^2^ | 63 | 2 | 0.5 |  | NSA |  |  |  |  |
| Left medial temporal lobe | Cho/Cr ratio | Jessen, 2001^3^ | 13 | 2 | 1.9 | R_s_ = 0.58* | R_s_ = -0.39◘ |  |  |  |  |
|  | NAA/Cr ratio | Jessen, 2006^4^ | 17 | 2 | 0.2 |  | NSA |  |  |  |  |
|  | NAA/Cr ratio | Jessen, 2001^3^ | 13 | 2 | 1.9 | R_s_ = 0.77** | R_s_ = -0.56* |  |  |  |  |
|  | NAA conc. | Jessen, 2006^4^ | 17 | 2 | 0.2 |  | NSA |  |  |  |  |
| Left central region‡ | NAA/Cr ratio | Jessen, 2001^3^ | 13 | 2 | 1.9 | NSA | NSA |  |  |  |  |
|  | Cho/Cr ratio | Jessen, 2001^3^ | 13 | 2 | 1.9 | NSA | NSA |  |  |  |  |
| Left medial temporal lobe/left central region§ | NAA/Cr ratio | Jessen, 2001^3^ | 13 | 2 | 1.9 | NSA | NSA |  |  |  |  |
|  | Cho/Cr ratio | Jessen, 2001^3^ | 13 | 2 | 1.9 | NSA | NSA |  |  |  |  |

|  | | | |  |  | **Association of change in feature measured with change in:** | | | | | |
| --- | --- | --- | --- | --- | --- | --- | --- | --- | --- | --- | --- |
| **Region of brain where feature measured** | **Feature measured** | **Reference**  **(first author, year)** | **n at baseline** | **Number of scans** | **Time between first and last scan (years)** | **MMSE** | **ADAS-cog** | **ADAS-noncog** | **CDR-SB** | **DRS** | **IDDD** |
| Left parietal lobe | NAA/Cr ratio | Jessen, 2006^4^ | 17 | 2 | 0.2 |  | R = -0.69** |  |  |  |  |
|  | NAA conc. | Jessen, 2006^4^ | 17 | 2 | 0.2 |  | R = -0.63* |  |  |  |  |
| Right midparietal cortex | NAA/Cr ratio | Modrego, 2006^1^ | 24 | 2 | 0.3 |  | NSA | NSA |  |  | NSA |
|  | mI/Cr ratio | Modrego, 2006^1^ | 24 | 2 | 0.3 |  | NSA | NSA |  |  | NSA |
| Midline bilateral occipital cortex | NAA/Cr ratio | Modrego, 2006^1^ | 24 | 2 | 0.3 |  | NSA | NSA |  |  | NSA |
|  | mI/Cr ratio | Modrego, 2006^1^ | 24 | 2 | 0.3 |  | NSA | NSA |  |  | NSA |
| Left medial occipital lobe | NAA/Cr ratio | Modrego, 2010^2^ | 63 | 2 | 0.5 |  | NSA |  |  |  |  |
| Posterior cingulate gyri and inferior precunei† | NAA/Cr ratio | Kantarci, 2007^5^ | 60 | 2 | 1.1 | R_s_ = -0.16 ◘ |  |  | R_s_ = -0.30* | R_s_ = 0.39** |  |
|  | Cho/Cr ratio | Kantarci, 2007^5^ | 60 | 2 | 1.1 | R_s_ = -0.32* |  |  | R_s_ = 0.01◘ | R_s_ = -0.08◘ |  |
| Bilateral posterior cingulate gyrus | NAA/Cr ratio | Modrego, 2010^2^ | 63 | 2 | 0.5 |  | R_?_ = -0.36** |  |  |  |  |
| Right hippocampus | mI/Cho ratio | Bartha, 2008^6^ | 10 | 2 | 0.3 |  | R_?_ = 0.59◘ |  |  |  |  |
|  | Cho/Cr ratio | Bartha, 2008^6^ | 10 | 2 | 0.3 | R_?_ = -0.62◘ |  |  |  |  |  |

|  | | | |  |  | **Association of change in feature measured with change in:** | | | | | |
| --- | --- | --- | --- | --- | --- | --- | --- | --- | --- | --- | --- |
| **Region of brain where feature measured** | **Feature measured** | **Reference**  **(first author, year)** | **n at baseline** | **Number of scans** | **Time between first and last scan (years)** | **MMSE** | **ADAS-cog** | **ADAS-noncog** | **CDR-SB** | **DRS** | **IDDD** |
| Grey matter | NAA conc. | Adalsteinsson, 2000^7^ | 12 | 2 | 0.9 |  |  |  |  | R_?_ = 0.54* |  |
| Cortical grey matter | NAA conc. | Krishnan, 2003^8^ | 33 | 6 | 0.6 |  | NSA |  |  |  |  |
| Subcortical grey matter | NAA conc. | Krishnan, 2003^8^ | 33 | 6 | 0.6 |  | R_?_ = 0.42* |  |  |  |  |
| Periventricular grey matter | NAA conc. | Krishnan, 2003^8^ | 33 | 6 | 0.6 |  | NSA |  |  |  |  |
| White matter | NAA conc. | Krishnan, 2003^8^ | 33 | 6 | 0.6 |  | NSA |  |  |  |  |

**Key**


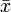
 Where this symbol is show then the value given is the average of left and right hemispheric structures. If not shown then it is unclear from the text whether the value represents an average or a total (left and right hemispheric structures combined) value.

Cho Choline

Conc. Absolute concentration

Cr Creatine

MRS Magnetic Resonance Spectroscopy

mI Myo-inositol

NAA N-acetylcysteine

Superscript numbers correspond to the list of references

**Correlations**

Pearson’s correlation coefficient R

Spearman’s correlation coefficient R_s_

Correlation coefficient unspecified R_?_

NSA No significant association No symbol: P not significant, but actual value not stated

POS Significant positive association ◘ P ≥ 0.05

NEG Significant negative association ^(^*^)^ P significant, but actual value not stated

SIG Significant association direction not stated * P < 0.05

** P < 0.01

*** P < 0.001

**Clinical Rating Scales**

ADAS-cog Alzheimer’s Disease Assessment Scale – cognitive subscale^9^

ADAS-noncog Alzheimer’s Disease Assessment Scale – non-cognitive subscale^9^

CDR-SB The Washington University Clinical Dementia Rating Sum-of-Boxes score^10^

DRS Dementia Rating Scale^11^

IDDD Interview for Deterioration in Daily living activities in Dementia^12^

MMSE Mini-Mental State Examination^13^

**References**

1. Modrego PJ, Pina MA, Fayed N, Diaz M (2006) Changes in metabolite ratios after treatment with rivastigmine in Alzheimer's disease: A nonrandomised controlled trial with magnetic resonance spectroscopy. CNS Drugs 20: 867-877.

2. Modrego PJ, Fayed N, Errea JM, Rios C, Pina MA, et al. (2010) Memantine versus donepezil in mild to moderate Alzheimer's disease: A randomized trial with magnetic resonance spectroscopy. Eur J Neurol 17: 405-412.

3. Jessen F, Block W, Traber F, Keller E, Flacke S, et al. (2001) Decrease of N-acetylaspartate in the MTL correlates with cognitive decline of AD patients. Neurology 57: 930-932.

4. Jessen F, Traeber F, Freymann K, Maier W, Schild HH, et al. (2006) Treatment monitoring and response prediction with proton MR spectroscopy in AD. Neurology 67: 528-530.

5. Kantarci K, Weigand SD, Petersen RC, Boeve BF, Knopman DS, et al. (2007) Longitudinal 1H MRS changes in mild cognitive impairment and Alzheimer's disease. Neurobiol Aging 28: 1330-1339.

6. Bartha R, Smith M, Rupsingh R, Rylett J, Wells JL, et al. (2008) High field (1)H MRS of the hippocampus after donepezil treatment in Alzheimer disease. Prog Neuropsychopharmacol Biol Psychiatry 32: 786-793.

7. Adalsteinsson E, Sullivan EV, Kleinhans N, Spielman DM, Pfefferbaum A (2000) Longitudinal decline of the neuronal marker N-acetyl aspartate in Alzheimer's disease. Lancet 355: 1696-1697.

8. Krishnan KR, Charles HC, Doraiswamy PM, Mintzer J, Weisler R, et al. (2003) Randomized, placebo-controlled trial of the effects of donepezil on neuronal markers and hippocampal volumes in Alzheimer's disease. Am J Psychiatry 160: 2003-2011.

9. Mohs RC, Knopman D, Petersen RC, Ferris SH, Ernesto C, et al. (1997) Development of cognitive instruments for use in clinical trials of antidementia drugs: additions to the Alzheimer's Disease Assessment Scale that broaden its scope. The Alzheimer's Disease Cooperative Study. Alzheimer Dis Assoc Disord 11: S13-S21.

10. Morris JC (1993) The Clinical Dementia Rating (CDR): current version and scoring rules. Neurology 43: 2412-2414.

11. Brown GG, Rahill AA, Gorell JM, McDonald C, Brown SJ, et al. (1999) Validity of the Dementia Rating Scale in assessing cognitive function in Parkinson's disease. J Geriatr Psychiatry Neurol 12: 180-188.

12. Teunisse S, Derix MM (1997) The interview for deterioration in daily living activities in dementia: agreement between primary and secondary caregivers. Int Psychogeriatr 9: 155-162.

13. Folstein MF, Folstein SE, McHugh PR (1975) "Mini-mental state". A practical method for grading the cognitive state of patients for the clinician. J Psychiatr Res 12: 189-198.
