# Supplementary material for: A Systematic Review of Biomarkers for Disease Progression in Alzheimer's Disease
Source: PLoS One. 2014 Feb 18;9(2):e88854. doi: 10.1371/journal.pone.0088854 (PMC3928315; doi:10.1371/journal.pone.0088854)
Supplement: Table S7 — Brain SPECT biomarkers. (DOCX) [file pone.0088854.s009.docx]

# Table S7 *Brain SPECT*

**Associations between putative brain SPECT biomarkers and clinical measures of disease severity, in longitudinal studies included in the systemic review of biomarkers for disease progression in Alzheimer’s disease**

|  | | | |  |  | **Association of change in ligand binding with change in:** | | | | |
| --- | --- | --- | --- | --- | --- | --- | --- | --- | --- | --- |
| **SPECT ligand** | **Region in which change in binding measured** | **Reference**  **(first author, year)** | **n at baseline** | **Number of scans** | **Time between first and last scan (years)** | **MMSE** | **GNFI** | **Blessed IMC** | **Total CAMCOG** | **CAMCOG (memory)** |
| [^123^I]IMP | Frontal lobe |  |  |  |  |  |  |  |  |  |
|  | Right | Shimizu, 2006^1^ | 51 | 2 | 1.0 | R_s_ = -0.132 |  |  |  |  |
|  | Left | Shimizu, 2006^1^ | 51 | 2 | 1.0 | R_s_ = -0.391* |  |  |  |  |
|  | Temporal lobe |  |  |  |  |  |  |  |  |  |
|  | Right | Shimizu, 2006^1^ | 51 | 2 | 1.0 | R_s_ = -0.182 |  |  |  |  |
|  | Left | Shimizu, 2006^1^ | 51 | 2 | 1.0 | R_s_ = -0.267 |  |  |  |  |
|  | Parietal lobe |  |  |  |  |  |  |  |  |  |
|  | Right | Shimizu, 2006^1^ | 51 | 2 | 1.0 | R_s_ = -0.295 |  |  |  |  |
|  | Left | Shimizu, 2006^1^ | 51 | 2 | 1.0 | R_s_ = -0.239 |  |  |  |  |
|  | Occipital lobe |  |  |  |  |  |  |  |  |  |
|  | Right | Shimizu, 2006^1^ | 51 | 2 | 1.0 | R_s_ = -0.046 |  |  |  |  |
|  | Left | Shimizu, 2006^1^ | 51 | 2 | 1.0 | R_s_ = -0.043 |  |  |  |  |
|  | Limbic lobe |  |  |  |  |  |  |  |  |  |
|  | Right | Shimizu, 2006^1^ | 51 | 2 | 1.0 | R_s_ = -0.241 |  |  |  |  |
|  | Left | Shimizu, 2006^1^ | 51 | 2 | 1.0 | R_s_ = -0.441** |  |  |  |  |
| HMPAO | Frontal | Sachdev, 1997^2^ | 27 | 2 | 2.0 | R_s_ = 0.69* | R_s_ = 0.16 | R_s_ = 0.21 |  |  |
|  | Low frontal |  |  |  |  |  |  |  |  |  |
|  | Right | Brown, 1996^3^ | 24 | 2 | 2.0 |  |  |  | R_?_ = 0.48*† | NSA◘ |
|  | Left | Brown, 1996^3^ | 24 | 2 | 2.0 |  |  |  | R_?_ = 0.55*† | NSA◘ |

|  | | | |  |  | **Association of change in ligand binding with change in:** | | | | |
| --- | --- | --- | --- | --- | --- | --- | --- | --- | --- | --- |
| **SPECT ligand** | **Region in which change in binding measured** | **Reference**  **(first author, year)** | **n at baseline** | **Number of scans** | **Time between first and last scan (years)** | **MMSE** | **GNFI** | **Blessed IMC** | **Total CAMCOG** | **CAMCOG (memory)** |
| HMPAO  (cont.) | High frontal |  |  |  |  |  |  |  |  |  |
|  | Right | Brown, 1996^3^ | 24 | 2 | 2.0 |  |  |  | NSA◘ | NSA◘ |
|  | Left | Brown, 1996^3^ | 24 | 2 | 2.0 |  |  |  | NSA◘ | NSA◘ |
|  | Temporal |  |  |  |  |  |  |  |  |  |
|  | Right | Brown, 1996^3^ | 24 | 2 | 2.0 |  |  |  | R_?_ = 0.39*† | NSA◘ |
|  |  | Sachdev, 1997^2^ | 27 | 2 | 2.0 | R_s_ = 0.55 | R_s_ = 0.55 | R_s_ = 0.09 |  |  |
|  | Left | Brown, 1996^3^ | 24 | 2 | 2.0 |  |  |  | NSA◘ | NSA◘ |
|  |  | Sachdev, 1997^2^ | 27 | 2 | 2.0 | R_s_ = 0.57* | R_s_ = 0.30 | R_s_ = 0.21 |  |  |
|  | Posterior temporal |  |  |  |  |  |  |  |  |  |
|  | Right | Brown, 1996^3^ | 24 | 2 | 2.0 |  |  |  | NSA◘ | NSA◘ |
|  | Left | Brown, 1996^3^ | 24 | 2 | 2.0 |  |  |  | NSA◘ | NSA◘ |
|  | Left frontal-temporal region‡ | Nobili, 2002^4^ | 25 | 2 | 0.9 | POS* |  |  |  |  |
|  | Parietal |  |  |  |  |  |  |  |  |  |
|  | Right | Brown, 1996^3^ | 24 | 2 | 2.0 |  |  |  | NSA◘ | NSA◘ |
|  | Left | Brown, 1996^3^ | 24 | 2 | 2.0 |  |  |  | NSA◘ | NSA◘ |
|  | Entire cerebrum | Sachdev, 1997^2^ | 27 | 2 | 2.0 | R_s_ = 0.19 | R_s_ = 0.28 | R_s_ = 0.19 |  |  |
|  | Hemisphere |  |  |  |  |  |  |  |  |  |
|  | Right | Sachdev, 1997^2^ | 27 | 2 | 2.0 | R_s_ = 0.29 | R_s_ = 0.11 | R_s_ = 0.41 |  |  |
|  | Left | Sachdev, 1997^2^ | 27 | 2 | 2.0 | R_s_ = 0.21 | R_s_ = 0.33 | R_s_ = 0.11 |  |  |
|  | 25 different brain regions§ | Cerci, 2007^5^ | 15 | 2 | 0.5 | NSA◘ |  |  |  |  |

**Key**

† It is unclear whether the stated values are Pearson correlation coefficients or R^2^ values.

‡ Statistical parametric mapping (SPM) and analysis of covariance (ANCOVA) showed that the change in MMSE over time in this study was significantly correlated with the change in regional cerebral blood flow in a large frontal-temporal region of the left hemisphere, with a maximum correlation in the left uncus and amygdala.

§ This paper stated that there was no correlation between the change in MMSE and the change in regional cerebral blood flow, as measured by HMPAO SPECT, in the study group. Earlier in the methodology it is stated that 25 different cortical regions were examined. It is, therefore, assumed that this statement refers to these 25 regions.


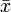
 Where this symbol is show then the value given is the average of left and right hemispheric structures. If not shown then it is unclear from the text whether the value represents an average or a total (left and right hemispheric structures combined) value.

**SPECT ligands**

[^123^I]IMP N-isopropyl-P[^123^I]-iodoamphetamine

HMPAO [^99m^Tc]-hexamethylpropylene amine oxidase

Superscript numbers correspond to the list of references

**Correlations**

Spearman’s correlation coefficient R_s_

Correlation coefficient unspecified R_?_

NSA No significant association No symbol: P not significant, but actual value not stated

POS Significant positive association ◘ P ≥ 0.05

NEG Significant negative association ^(^*^)^ P significant, but actual value not stated

SIG Significant association direction not stated * P < 0.05

** P < 0.01

*** P < 0.001

**Clinical Rating Scales**

Blessed IMC Blessed Dementia Information-Memory-Concentration test^6^

CAMCOG (memory) The memory subsection of the Cambridge Examination for Mental disorders of the Elderly^7^

GNFI Global Neuropsychological Function Index^2^

MMSE Mini-Mental State Examination^8^

Total CAMCOG The cognitive and self-contained part of the Cambridge Examination for Mental disorders of the Elderly^7^

**References**

1. Shimizu S, Hanyu H, Iwamoto T, Koizumi K, Abe K (2006) SPECT follow-up study of cerebral blood flow changes during Donepezil therapy in patients with Alzheimer's disease. J Neuroimaging 16: 16-23.

2. Sachdev P, Gaur R, Brodaty H, Walker A, Meares S, et al. (1997) Longitudinal study of cerebral blood flow in Alzheimer's disease using single photon emission tomography. Psychiatry Res 68: 133-141.

3. Brown DRP, Hunter R, Wyper DJ, Patterson J, Kelly RC, et al. (1996) Longitudinal changes in cognitive function and regional cerebral function in Alzheimer's disease: A spect blood flow study. J Psychiatr Res 30: 109-126.

4. Nobili F, Vitali P, Canfora M, Girtler N, De LC, et al. (2002) Effects of long-term Donepezil therapy on rCBF of Alzheimer's patients. Clin Neurophysiol 113: 1241-1248.

5. Cerci SS, Tamam Y, Kaya H, Yildiz M, Arslan S (2007) Effect of rivastigmine on regional cerebral blood flow in Alzheimer's disease. Adv Ther 24: 611-621.

6. Blessed G, Tomlinson BE, Roth M (1968) The association between quantitative measures of dementia and of senile change in the cerebral grey matter of elderly subjects. Br J Psychiatry 114: 797-811.

7. Roth M, Tym E, Mountjoy CQ, Huppert FA, Hendrie H, et al. (1986) CAMDEX. A standardised instrument for the diagnosis of mental disorder in the elderly with special reference to the early detection of dementia. Br J Psychiatry 149: 698-709.

8. Folstein MF, Folstein SE, McHugh PR (1975) "Mini-mental state". A practical method for grading the cognitive state of patients for the clinician. J Psychiatr Res 12: 189-198.
