# Supplementary material for: A Systematic Review of Biomarkers for Disease Progression in Alzheimer's Disease
Source: PLoS One. 2014 Feb 18;9(2):e88854. doi: 10.1371/journal.pone.0088854 (PMC3928315; doi:10.1371/journal.pone.0088854)
Supplement: Table S8 — Brain PET biomarkers. (DOCX) [file pone.0088854.s010.docx]

# Table S8 *Brain PET*

**Associations between putative brain PET biomarkers and clinical measures of disease severity, in longitudinal studies included in the systemic review of biomarkers for disease progression in Alzheimer’s disease**

|  | | | |  |  | **Association of change in ligand binding with change in:** | | |
| --- | --- | --- | --- | --- | --- | --- | --- | --- |
| **PET ligand** | **Region in which change in binding measured** | **Reference**  **(first author, year)** | **n at baseline** | **Number of scans** | **Time between first and last scan (years)** | **MMSE** | **ADAS-cog** | **CDR-SB** |
| FDG | Frontal cortex | Engler, 2006^1^ | 16 | 2 | 1.9 | NSA |  |  |
|  |  | Mielke, 1994^2^ | 25 | 2 | 1.1 | R = 0.4◘ |  |  |
|  | Occipital cortex | Engler, 2006^1^ | 16 | 2 | 1.9 | NSA |  |  |
|  |  | Mielke, 1994^2^ | 25 | 2 | 1.1 | R = 0.44* |  |  |
|  | Parietal cortex | Engler, 2006^1^ | 16 | 2 | 1.9 | R = 0.59* |  |  |
|  | Temporal cortex | Engler, 2006^1^ | 16 | 2 | 1.9 | NSA |  |  |
|  | Temporoparietal cortex | Mielke, 1994^2^ | 25 | 2 | 1.1 | R = 0.49* |  |  |
|  | Striatum | Engler, 2006^1^ | 16 | 2 | 1.9 | NSA |  |  |
|  | Subcortical white matter | Engler, 2006^1^ | 16 | 2 | 1.9 | NSA |  |  |
|  | Cerebellum cortex | Engler, 2006^1^ | 16 | 2 | 1.9 | NSA |  |  |
|  | Pons | Engler, 2006^1^ | 16 | 2 | 1.9 | NSA |  |  |
|  | Entire cerebrum | Mielke, 1994^2^ | 25 | 2 | 1.1 | R = 0.38 |  |  |

|  | | | |  |  | **Association of change in ligand binding with change in:** | | |
| --- | --- | --- | --- | --- | --- | --- | --- | --- |
| **PET ligand** | **Region in which change in binding measured** | **Reference**  **(first author, year)** | **n at baseline** | **Number of scans** | **Time between first and last scan (years)** | **MMSE** | **ADAS-cog** | **CDR-SB** |
| FDG  (continued) | Left cingulate | Mega, 2005^3^ | 12 | 2 | 0.2 | R = 0.70* |  |  |
|  | Metabolic rate† | Mielke, 1994^2^ | 25 | 2 | 1.1 | R = 0.28 |  |  |
|  | PET score‡ | Herholz, 2011^4^ | 35 | 4 | 2.0 |  |  |  |
|  | Statistical region-of-interest§ | Chen, 2010^5^ | 69 | 2 | 1.0 | R_?_ = 0.23◘ | R_?_ = 0.59 | R_?_ = 0.25* |
| PiB | Frontal cortex | Engler, 2006^1^ | 16 | 2 | 1.9 | NSA |  |  |
|  | Occipital cortex | Engler, 2006^1^ | 16 | 2 | 1.9 | NSA |  |  |
|  | Parietal cortex | Engler, 2006^1^ | 16 | 2 | 1.9 | NSA |  |  |
|  | Temporal cortex | Engler, 2006^1^ | 16 | 2 | 1.9 | NSA |  |  |
|  | Striatum | Engler, 2006^1^ | 16 | 2 | 1.9 | NSA |  |  |
|  | Subcortical white matter | Engler, 2006^1^ | 16 | 2 | 1.9 | NSA |  |  |
|  | Cerebellum cortex | Engler, 2006^1^ | 16 | 2 | 1.9 | NSA |  |  |
|  | Pons | Engler, 2006^1^ | 16 | 2 | 1.9 | NSA |  |  |
|  | Neocortical Aβ burden (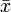)• | Villemagne, 2011^6^ | 35 | 3 | 3.3 | NSA |  |  |

**Key**

† The ‘metabolic rate’ equals the mean regional cerebral glucose metabolism in regions typically affected by Alzheimer’s disease (temporoparietal and frontal association cortex) divided by the mean regional cerebral glucose metabolism in unaffected regions (cerebellum, brainstem, lentiform nucleus, primary visual and sensorimotor cortex).

‡ PET score = log2 [(ADtsum/11089) + 1]. AD t-sum indicates the severity of the metabolic decrease in brain areas that are typically affected by Alzheimer’s disease (multimodal association cortices mostly located in the temporal and parietal lobes), including an adjustment for age.

§ This study examined the change in the regional cerebral metabolic rate for glucose in a pre-defined statistical region-of-interest. This included voxels consistently associated with decline as determined by the application of statistical parametric mapping (SPM) to a training data set.

• Neocortial Aβ burden was expressed as the average standardised uptake values (SUVR) of the area-weighted mean (i.e. averaged across both hemispheres) of frontal, superior parietal, lateral temporal, lateral occipital, and anterior and posterior cingulated regions.


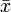
 Where this symbol is show then the value given is the average of left and right hemispheric structures. If not shown then it is unclear from the text whether the value represents an average or a total (left and right hemispheric structures combined) value.

**PET ligands**

FDG [^18^F]-2-fluoro-2-deoxyglucose

PiB [^11^C]Pittsburgh compound B

Superscript numbers correspond to the list of references

**Correlations**

Pearson’s correlation coefficient R

Correlation coefficient unspecified R_?_

NSA No significant association No symbol: P not significant, but actual value not stated

POS Significant positive association ◘ P ≥ 0.05

NEG Significant negative association ^(^*^)^ P significant, but actual value not stated

SIG Significant association direction not stated * P < 0.05

** P < 0.01

*** P < 0.001

**Clinical Rating Scales**

ADAS-cog Alzheimer’s Disease Assessment Scale – cognitive subscale^7^

CDR-SB The Washington University Clinical Dementia Rating Sum-of-Boxes score^8^

MMSE Mini-Mental State Examination^9^

**References**

1. Engler H, Forsberg A, Almkvist O, Blomquist G, Larsson E, et al. (2006) Two-year follow-up of amyloid deposition in patients with Alzheimer's disease. Brain 129: 2856-2866.

2. Mielke R, Herholz K, Grond M, Kessler J, Heiss WD (1994) Clinical deterioration in probable Alzheimer's disease correlates with progressive metabolic impairment of association areas. Dementia 5: 36-41.

3. Mega MS, Dinov ID, Porter V, Chow G, Reback E, et al. (2005) Metabolic patterns associated with the clinical response to galantamine therapy: a fludeoxyglucose f 18 positron emission tomographic study. Arch Neurol 62: 721-728.

4. Herholz K, Westwood S, Haense C, Dunn G (2011) Evaluation of a calibrated 18F-FDG PET score as a biomarker for progression in alzheimer disease and mild cognitive impairment. J Nucl Med 52: 1218-1226.

5. Chen K, Langbaum JB, Fleisher AS, Ayutyanont N, Reschke C, et al., Alzheimer's Disease Neuroimaging Initiative (2010) Twelve-month metabolic declines in probable Alzheimer's disease and amnestic mild cognitive impairment assessed using an empirically pre-defined statistical region-of-interest: findings from the Alzheimer's Disease Neuroimaging Initiative. Neuroimage 51: 654-664.

6. Villemagne VL, Pike KE, Chetelat G, Ellis KA, Mulligan RS, et al. (2011) Longitudinal assessment of Abeta and cognition in aging and Alzheimer disease. Ann Neurol 69: 181-192.

7. Mohs RC, Knopman D, Petersen RC, Ferris SH, Ernesto C, et al. (1997) Development of cognitive instruments for use in clinical trials of antidementia drugs: additions to the Alzheimer's Disease Assessment Scale that broaden its scope. The Alzheimer's Disease Cooperative Study. Alzheimer Dis Assoc Disord 11: S13-S21.

8. Morris JC (1993) The Clinical Dementia Rating (CDR): current version and scoring rules. Neurology 43: 2412-2414.

9. Folstein MF, Folstein SE, McHugh PR (1975) "Mini-mental state". A practical method for grading the cognitive state of patients for the clinician. J Psychiatr Res 12: 189-198.
