# Supplementary material for: A Systematic Review of Biomarkers for Disease Progression in Alzheimer's Disease
Source: PLoS One. 2014 Feb 18;9(2):e88854. doi: 10.1371/journal.pone.0088854 (PMC3928315; doi:10.1371/journal.pone.0088854)
Supplement: Table S9 — Electrophysiological biomarkers. (DOCX) [file pone.0088854.s011.docx]

# Table S9 *Electrophysiology*

**Associations between putative electrophysiological biomarkers and clinical measures of disease severity, in longitudinal studies included in the systemic review of biomarkers for disease progression in Alzheimer’s disease**

| **Electrophysiological** | |  | | | | **Association of change in feature measured with change in:** | | | |
| --- | --- | --- | --- | --- | --- | --- | --- | --- | --- |
| **Modality** | **Feature measured** | **Reference**  **(first author, year)** | **n at baseline** | **Number of sampling intervals** | **Time between first and last samples (years)** | **MMSE** | **ADAS-cog** | **Global CDR** | **CDR-SB** |
| Electroencephalography (EEG) at rest | Delta power | Babiloni, 2009^1^ | 23 | 2 | 1.0 | NSA |  | R_s_ = -0.5* |  |
| Electrooculography (EOG) | Number of intrusive saccades | Bylsma, 1995^2^ | 17 | 2 | 0.8 | R = -0.42* |  |  |  |
|  | Latency of initiation of saccade |  |  |  |  |  |  |  |  |
|  | target amplitude 7˚ | Bylsma, 1995^2^ | 17 | 2 | 0.8 | NSA |  |  |  |
|  | target amplitude 15˚ | Bylsma, 1995^2^ | 17 | 2 | 0.8 | NSA |  |  |  |
|  | Amplitude of saccade | Bylsma, 1995^2^ | 17 | 2 | 0.8 | NSA |  |  |  |
|  | target amplitude 7˚ | Bylsma, 1995^2^ | 17 | 2 | 0.8 | NSA |  |  |  |
|  | target amplitude 15˚ | Bylsma, 1995^2^ | 17 | 2 | 0.8 | NSA |  |  |  |
|  | Adjusted amplitude of saccade |  |  |  |  |  |  |  |  |
|  | target amplitude 7˚ | Bylsma, 1995^2^ | 17 | 2 | 0.8 | NSA |  |  |  |
|  | target amplitude 15˚ | Bylsma, 1995^2^ | 17 | 2 | 0.8 | NSA |  |  |  |
|  | Maximum velocity of saccade |  |  |  |  |  |  |  |  |
|  | target amplitude 7˚ | Bylsma, 1995^2^ | 17 | 2 | 0.8 | NSA |  |  |  |
|  | target amplitude 15˚ | Bylsma, 1995^2^ | 17 | 2 | 0.8 | NSA |  |  |  |

| **Electrophysiological** | |  | | | | **Association of change in feature measured with change in:** | | |
| --- | --- | --- | --- | --- | --- | --- | --- | --- |
| **Modality** | **Feature measured** | **Reference**  **(first author, year)** | **n at baseline** | **Number of sampling intervals** | **Time between first and last samples (years)** | **MMSE** | **ADAS-cog** | **CDR-SB** |
| Paired-pulse transcranial magnetic stimulation (pTMS) | Cortical excitability at 13 interstimulus intervals† | Olazarán, 2010^3^ |  |  |  | NSA | NSA |  |
| Magnetoencephalography (MEG) | cO^2^ values  (20910 sensor pairs)‡ | Verdoorn, 2011^4^ | 31 | 2 | 0.8 | R range:  -0.52 to -0.53  The change in 10% of total sensor pairs showed a significant correlation with the change in MMSE | R range:  -0.67 to -0.79  The change in 12% of total sensor pairs showed a significant correlation with the change in ADAS-cog | R range  -0.62 to -0.62  The change in 10% of total sensor pairs showed a significant correlation with the change in CDR-SB |
|  | Centroid frequency  (205 sensors)§ | Verdoorn, 2011^4^ | 31 | 2 | 0.8 | R range:  -0.47 to -0.27.  The change in 16% of sensors showed a significant correlation with the change in MMSE | R range:  -0.43 to -0.01  The change in 13% of sensors showed a significant correlation with the change in ADAS-cog | R range:  -0.47 to -0.18  The change in 23% of sensors showed a significant correlation with the change in CDR-SB |

**Key**

† Used 13 interstimulus intervals (ISIs) ranging from 1 to 300ms (1,2,3,5,10,12,14,16,20,30,80,100,300ms).

‡ cO is a zero-lag correlation value which includes information about direction and magnitude of correlated activity. In essence it is a measure of functional connectivity between different brain areas. In this study the authors state that in most cases a comparison of magnitude rather than direction was more informative and relevant. Therefore, they used the square of cO, cO^2^.

§ Centroid frequency is a measure of the distribution of relative spectral power.

Superscript numbers correspond to the list of references

**Correlations**

Pearson’s correlation coefficient R

Spearman’s correlation coefficient R_s_

NSA No significant association No symbol: P not significant, but actual value not stated

POS Significant positive association ◘ P ≥ 0.05

NEG Significant negative association ^(^*^)^ P significant, but actual value not stated

SIG Significant association direction not stated * P < 0.05

** P < 0.01

*** P < 0.001

**Clinical Rating Scales**

ADAS-cog Alzheimer’s Disease Assessment Scale – cognitive subscale^5^

CDR-SB The Washington University Clinical Dementia Rating Sum-of-Boxes score^6^

Global CDR The Washington University Clinical Dementia Rating global score^6^

MMSE Mini-Mental State Examination^7^

**References**

1. Babiloni C, Frisoni GB, Del PC, Zanetti O, Bonomini C, et al. (2009) Ibuprofen treatment modifies cortical sources of EEG rhythms in mild Alzheimer's disease. Clin Neurophysiol 120: 709-718.

2. Bylsma FW (1995) Changes in visual fixation and saccadic eye movements in Alzheimer's disease. Int Journal Psychophysiol 19: 33-40.

3. Olazaran J, Prieto J, Cruz I, Esteban A (2010) Cortical excitability in very mild Alzheimer's disease: A long-term follow-up study. J Neurol 257: 2078-2085.

4. Verdoorn TA, McCarten JR, Arcienegas DB, Golden R, Moldauer L, et al. (2011) Evaluation and tracking of Alzheimer's disease severity using resting-state magnetoencephalography. J Alzheimers Dis 26: 239-255.

5. Mohs RC, Knopman D, Petersen RC, Ferris SH, Ernesto C, et al. (1997) Development of cognitive instruments for use in clinical trials of antidementia drugs: additions to the Alzheimer's Disease Assessment Scale that broaden its scope. The Alzheimer's Disease Cooperative Study. Alzheimer Dis Assoc Disord 11: S13-S21.

6. Morris JC (1993) The Clinical Dementia Rating (CDR): current version and scoring rules. Neurology 43: 2412-2414.

7. Folstein MF, Folstein SE, McHugh PR (1975) "Mini-mental state". A practical method for grading the cognitive state of patients for the clinician. J Psychiatr Res 12: 189-198.
